# Chloroplast Genome Sequencing, Comparative Analysis, and Discovery of Unique Cytoplasmic Variants in Pomegranate (*Punica granatum* L.)

**DOI:** 10.3389/fgene.2021.704075

**Published:** 2021-07-28

**Authors:** Nripendra Vikram Singh, Prakash Goudappa Patil, Roopa P. Sowjanya, Shilpa Parashuram, Purushothaman Natarajan, Karuppannan Dhinesh Babu, Ram Krishna Pal, Jyotsana Sharma, Umesh K. Reddy

**Affiliations:** ^1^ICAR-National Research Centre on Pomegranate (NRCP), Solapur, India; ^2^Gus R. Douglass Institute and Department of Biology, West Virginia State University, West Virginia, WV, United States

**Keywords:** chloroplast, plastid genome, pomegranate, phylogenetic, sequence diversity

## Abstract

Here we report on comprehensive chloroplast (cp) genome analysis of 16 pomegranate (*Punica granatum* L.) genotypes representing commercial cultivars, ornamental and wild types, through large-scale sequencing and assembling using next-generation sequencing (NGS) technology. Comparative genome analysis revealed that the size of cp genomes varied from 158,593 bp (in wild, “1201” and “1181”) to 158,662 bp (cultivar, “Gul-e-Shah Red”) among the genotypes, with characteristic quadripartite structures separated by a pair of inverted repeats (IRs). The higher conservation for the total number of coding and non-coding genes (rRNA and tRNA) and their sizes, and IRs (IR-A and IR-B) were observed across all the cp genomes. Interestingly, high variations were observed in sizes of large single copy (LSC, 88,976 to 89,044 bp) and small single copy (SSC, 18,682 to 18,684 bp) regions. Although, the structural organization of newly assembled cp genomes were comparable to that of previously reported cp genomes of pomegranate (“Helow,” “Tunisia,” and “Bhagawa”), the striking differences were observed with the *Lagerstroemia* lines, viz., *Lagerstroemia intermedia* (NC_0346620) and *Lagerstroemia speciosa* (NC_031414), which clearly confirmed previous findings. Furthermore, phylogenetic analysis also revealed that members outside the genus *Punica* were clubbed into a separate clade. The contraction and expansion analysis revealed that the structural variations in IRs, LSC, and SSC have significantly accounted for the evolution of cp genomes of *Punica* and *L. intermedia* over the periods. Microsatellite survey across cp genomes resulted in the identification of a total of 233 to 234 SSRs, with majority of them being mono- (A/T or C/G, 164–165 numbers), followed by di- (AT/AT or AG/CT, 54), tri- (6), tetra- (8), and pentanucleotides (1). Furthermore, the comparative structural variant analyses across cp genomes resulted in the identification of many varietal specific SNP/indel markers. In summary, our study has offered a successful development of large-scale cp genomics resources to leverage future genetic, taxonomical, and phylogenetic studies in pomegranate.

## Introduction

Pomegranate (*Punica granatum* L.) is one of the oldest edible fruit crops belonging to the family Lythraceae (Dandachi et al., 2017). It is believed to have originated from Iran and is widely grown in drier parts of the world, i.e., Southeast Asia, India, Iran, China, Japan, United States (California), Israel, Spain, Turkey, and Israel (Yan et al., 2019a; Patil et al., 2020, 2021; Singh et al., 2020a). Being a native of central Asia that is from Iran to Western Himalayas in India, later, it was domesticated to many other parts of the world (Holland et al., 2009). Initially, pomegranate was placed under the family Punicaceae; later, based on morphological and molecular evidences, it was included in the family Lythraceae (Ii, 2003; Graham and Graham, 2014; Berger et al., 2016). In addition to having good economic value, pomegranate has excellent medicinal properties that made this crop an important cultivable fruit crop. Therefore, globally, this crop has gained tremendous popularity than other fruit crops. The inherent therapeutic and neutraceutical properties, better returns on investment in water scarce regions, appreciable export demand, and immense scope of value addition has made this crop as the chicest fruit for cultivation in semiarid tropics (Singh et al., 2021).

Pomegranate is popularly considered as a powerhouse of energy due to its immense nutritive value in terms of having many beneficial phytochemicals and antioxidant properties (Halvorsen et al., 2002). The edible and non-edible parts of the fruits have been reported to prevent and cure a wide range of human diseases including cancer, diabetes, obesity, Alzheimer’s disease, and hypertension (Seeram et al., 2006; Jalikop, 2010; Sharma and Maity, 2010; Singh et al., 2015, 2020b; Wang et al., 2015).

The genus *Punica* has two species, viz., *P. granatum* L. and *Punica protopunica* Balf.; of these, *P. protopunica* is considered as the ancestor of all cultivated pomegranate species and is confined to the region of the Socotra islands (Yemen). *P. protopunica* was believed to have contributed in the evolutionary process of the present-day cultivable pomegranate species (Chandra et al., 2010), which has short juvenile period with multiseeded fruits and, thus, making them best suited for performing genetic studies (da Silva et al., 2013; Yan et al., 2019b). Since, pomegranate has considerable variations within its species; the analysis of chloroplast and mitochondrial genomes of pomegranate genotypes would greatly help to understand the evolution of this crop at different taxonomic levels (Hao et al., 2010; Ruhfel et al., 2014; Sancho et al., 2018; Yan et al., 2019a).

With the recent advances in next-generation sequencing (NGS) technologies, now, it is possible to carryout whole chloroplast genome sequencing very quickly in a cost-effective manner (Khan et al., 2018a). In order to understand the evolution and phylogenetic relationship between several progenies, it is always better to go for the chloroplast genome analysis because it is haploid, maternally inherited, and possesses highly conserved genes (Khan et al., 2018a). Moreover, recently, the chloroplast genomics was found to be very promising and has been very widely used for phylogenetic studies, due to its single-parent inheritance (maternal) and recombination-free nature (Liu et al., 2018). The plant chloroplast genetics has greatly helped in understanding the gene flow, cytoplasmic diversity, and population differentiation (Diekmann et al., 2012; Zhang et al., 2017). Recently, chloroplasts reported to have many key roles in several plant processes such as plant defense, production of defense compounds, physiology and development, and alternative splicing of the transcripts (McFadden and van Dooren, 2004; Petrillo et al., 2014; Daniell et al., 2016; Toufexi et al., 2019).

Since, chloroplast genomes are found very informative in species identification, phylogenetic implications, and population genetic analyses (Yang et al., 2013). Our study represents the first of its kind, where we tried to report large-scale sequencing and assembling of chloroplast (cp) genomes of cultivars, ornamental, and wild types of pomegranate. Here, we report the successful development of first-hand information on cp genomics resources through comparative cp genome analysis leading to discovery of many varietal specific cytoplasmic variations like SNP/indels and SSR markers. This could leverage the future genomic-assisted breeding in pomegranate through genetic, taxonomical, and phylogenetic studies. Our study has also provided a greater insight into the comparative phylogenetic differentiation of the *Punica* genotypes along with other closely related species. This investigation has clearly reconfirmed the earlier findings that the very limited diversity for cp genome was witnessed within species; however, structural variations in inverted repeats (IRs), LSC, and SSC regions have significantly accounted for the evolution of *Punica* and *Lagerstromia* cp genomes resulting in cp genome size variations. This comprehensive study might be very useful in understanding the phylogenetic evolutionary history of pomegranate genotypes across the selected plant species.

## Materials and Methods

### Plant Material and DNA Extraction

In this study, 16 pomegranate genotypes representing 11 cultivars, 2 ornamental, and 3 wild types were selected (Table 1). The fresh leaves of these genotypes were collected from the pomegranate Field Gene Bank ICAR-NRC on Pomegranate, Solapur, India, located at geographical coordinates of 17°68′ N latitude, 75°91′ E longitude and at 483.5 m altitude from mean sea level. A modified CTAB protocol was used to extract the total genomic DNA (Doyle and Doyle, 1987), and the concentration and quality of extracted DNA were initially determined through gel electrophoresis.

**TABLE 1:** Pomegranate genotypes with their special features.

**Sl. no.**	**Name of variety/germplasm/hybrid**	**Code Pg v**	**Pedigree**	**Features**
1	Solapur Lal	SL	Hybrid derived from Bhagawa × [(Ganesh × Nana) × Daru]	Bio-fortified variety released form ICAR-NRCP, having high Zn, Fe, and ascorbic acid content, medium-sized fruits (200–250 g) with red rind and aril, seeds are medium hard
2	Solapur Anardana	SA	Hybrid derived from Bhagawa × [(Ganesh × Nana) × Daru]	Variety released form ICAR-NRCP suitable for *anaradana* preparation having medium-sized fruits, juice with 4.5% acidity
3	Bhagawa	B	Selection from the F_2_ population (Ganesh × Gul-e-Shah Red)	Commercial variety of India covers about 80% area under cultivation, having attractive red medium-sized fruits (250–300 g), soft seeded, medium to high yielding, less acidic, and sweet type
4	Double-flowering Type	DF	Ornamental petaloidy type, chromosome no. is 2n = 18	Ornamental purpose, seldom sets fruit as male parts get converted into petals
5	*Punica granatum* Nana	RN	Ornamental purpose	Separate species of *Punica granatum* sup sp. *Nana*, naturally dwarf, sets small fruits and flowers, has ornamental value, used in breeding program as moderately resistant to bacterial blight
6	Super Bhagawa	SB	Selection from Bhagawa	Commercial variety, attractive red medium-sized fruits (250–300 g), fruits mature 15 days earlier than Bhagawa
7	Mridula	M	Selection from the F_2_ population (Ganesh × Gul-e-Shah Red)	Deep red-colored aril and rind, sweet fruits, matures earlier than Bhagawa, fruit size smaller than Bhagawa
8	Wonderful	W	Leading variety of the World, originated in California	Red-colored rind and aril, large-sized fruit, hard seeded, many field variants have been selected from this variety and spread all across the world
9	Phule Arakta	A	Selection from the F_2_ population (Ganesh × Gul-e-Shah Red)	Deep red-colored aril and rind, sweet fruits, matures earlier than Bhagawa, fruit size smaller than Bhagawa
10	Ruby	R	Complex cross variety of ICAR-IIHR, [(Ganesh × Kabul) × Yercaud]—F1–F2 × (Ganesh × Gul-e-Shah Rose Pink)—F1–F2	Soft seeded, red rind and red arils, sweet juice, and medium-sized fruits
11	Jyoti	J	Seedling selection from Bassein Seedless and Dholka varieties	A pomegranate variety with soft seeds and pink aril has been released from the University of Agricultural Sciences, Bangalore
12	Ganesh	G	Selection made from Alandi	Pink-colored aril and yellow with red-tinged rind, fruits bigger than Bhagawa, sweet, and soft seeded
13	Gul-e-Shah Red	GR	Russian colored variety	Deep red-colored fruit, used in breeding program to enhance rind and aril color
14	IC-1201	1201	Wild type collected from western Himalayas	Tall trees with acidic fruits and moderately resistant to bacterial blight
15	IC-1181	1181	Wild type collected from western Himalayas	Tall trees with acidic fruits and moderately resistant to bacterial blight
16	IC-318718	8718	Wild type collected from western Himalayas	Tall trees with acidic fruits and moderately tolerant to fruit cracking

### Plastid Genome Sequencing

Primary QC was performed to assess the quality and quantity check of the extracted genomic DNA by using Qubit and Nanodrop facilities. The cp genome data were extracted from the raw reads generated through whole-genome sequencing, the Illumina paired-end sequencing was performed for all the pomegranate genotypes, whereas for the var. “Bhagawa,” additionally, the 10× Chromium-HiSeq X sequencing platform and SMRT Bell based PacBio Sequel platform were used for whole-genome sequencing. Library preparation was carried out by using the latest Illumina TruSeq Strand specific PCR free Library kit (Cat. No. 20015963), as per the instructions of the manufacturer. To start with the protocol, 1 μg of genomic DNA was used for Covaris shearing to generate three insert size ranges ∼180–250, ∼250–300, and ∼350--500 bp to prepare paired-end library. Libraries were sequenced using HiSeq 2500 platform to generate raw data, which were submitted to the Zenodo^1^.

### Assembly and Mapping of Plastid Genomes

Before proceeding for cp genome assembly, the quality control of the raw reads obtained from whole-genome sequencing was performed by using a FastQC pre-processor (Andrews, 2010). The raw sequencing data from the samples were hard trimmed for reads containing ambiguous bases, adapters, and low base qualities using Trimmomatic to retain the cleaned read lengths >36 bases (Bolger et al., 2014). Subsequently, *de novo* assembly of the plastid genome was accomplished using GetOrganelle *v.1.5.1c* (Jin et al., 2019). The plastid sequence of *P. granatum* (Acc: NC_035240) was taken as a bait to extract reads having respective organellar origin by mapping using Bowtie2 (Langdon, 2013). These reads were then assembled internally through GetOrganelle pipeline using SPAdes *de novo* assembler tool (Bankevich et al., 2012) and were further curated to evaluate the quality of assemblies using the script “slim_fastg_by_blast” and subsequently visualized as output fastg file in Bandage (Wick et al., 2015). The default parameters of these tools were used in every step of the *de novo* assembly.

### Annotation and Repeat Sequence Analysis

The web server CPGAVAS2 and GESeq were used for annotating and visualizing the plastid genomes (Tillich et al., 2017; Shi et al., 2019). We performed all the annotations using the 43 plastome datasets, which contain RNASeq-validated/-corrected sequences. Internally, BLAST runs on plastome sequences were performed using CPGAVAS2 to identify the gene models and rRNA genes, tRNAscan-se, ARAGON to develop a curated set of tRNAs, genes, and Vmatch to identify IR regions (Kurtz, 2003). These annotations were subsequently compiled using Maker to generate gff files (Campbell et al., 2014). In the case of GESeq, the chloroplast genome was annotated by selecting the options to perform HMMER profile search [chloroplast coding the DNA sequence (CDS) and rRNA], tRNAscan-SE, ARAGORN, and BLAST using the MPI-MP chloroplast references, while leaving all other default settings. In addition to this, the repeat pattern in the cp genomes were analyzed for simple and tandem repeats using MISA (“1–8 2–4 3–4 4–3 5–3 6–3”) (Thiel, 2003), dot2dot (Genovese et al., 2019), and TRF (motifs >7 bp) (Benson, 1999) search tools.

### Genome-Wide Structural Variant Analysis

All the 16 newly sequenced plastid genomes along with the published chloroplast genomes (NC035240.1 and MG878386.1) of pomegranate were used to identify the unique and conserved genes. For pan genome analysis, the Roary *v.3.11.2* software was used (Page et al., 2015). The structural variations across the shared genes of different pomegranate genotypes were resolved by using Assemblytics *v.1.0*. The Minimap2 (2_2.11) was used to align the assembled genomes against pomegranate chloroplast genome (NC035240.1) (Li et al., 2018), and variant calling was performed using Paftools (Ruan and Li, 2020). For variant calling analysis, ekidna (commit: 64e5d1c) was used (Nattestad and Schatz, 2016).

### Sequence Divergence-Based Phylogenetic Analysis

The ContigOrderer from Mauve was used to orient and order the all-sequenced and reported chloroplast genome of the multiple genotypes in accordance with the NCBI reference NC_035240.1 (Darling et al., 2004). To identify the divergent hotspots, all the ordered chloroplast genomes were aligned using the auto algorithm of MAFFT (v.7.407) (Katoh et al., 2009) at 1,000 iterations, and the rooted phylogenetic tree was drawn using the FastTreentgtr model to derive a newick tree at 1,000 bootstraps (Price et al., 2009). The newick tree was finally visualized using the iTol *v.3* online tool, by ignoring the branch lengths (Letunic and Bork, 2007).

## Results

### Organization and Gene Content of 16 *Punica* Plastid Genomes

**FIGURE 1:**
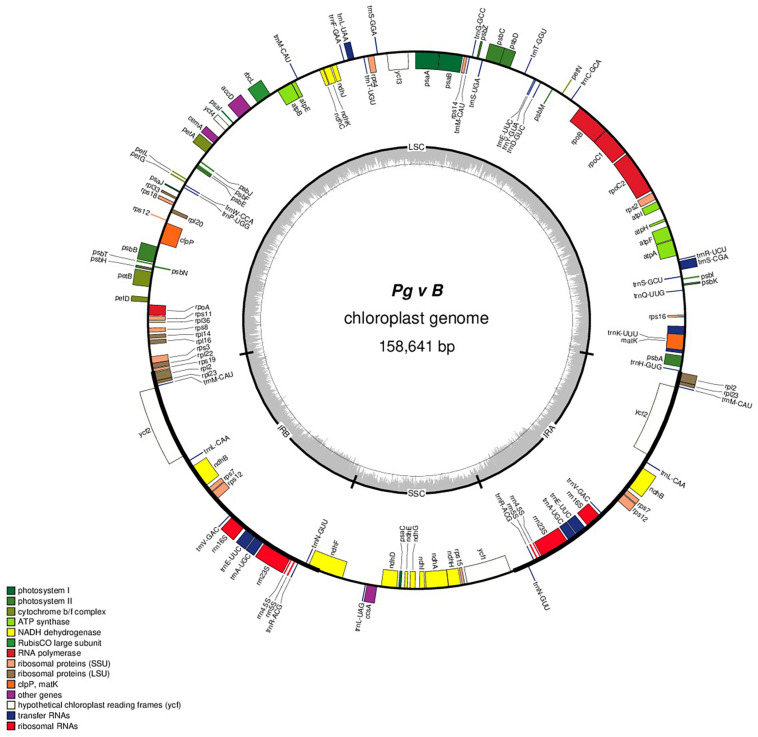
Genome map of the “Bhagawa” cp genome: The thick line in the outer ring indicates the extent of inverted repeat (IR) (IRA and IRB) regions. Genes drawn inside the circle are transcribed clockwise, and those outside are transcribed counter clockwise. Genes belonging to different functional groups are color coded. The dark gray in the inner circle corresponds to the GC content, and the light gray corresponds to the AT content.

The complete cp genomes of 16 *P. granatum* genotypes representing cultivars, ornamental, and wild types were sequenced through the Illumina paired-end sequencing and subsequently *de novo* assembly in SPAdes using the GetOrganelle pipeline and subsequently visualized in Bandage. The genomes ranged in size from 158,593 bp in wild (1201 and 1181) to 158,662 bp in cultivar (“Gul-e-Shah Red”), respectively (Table 2 and Supplementary File 1). All of these genomes exhibited a typical quadripartite structure with a pair of two IR repeats (25,467) separated by the large single-copy regions (LSC: 89,044–88,976) and a small single-copy regions (SSC: 18,682–18,685). The representative chloroplast genome map of pomegranate variety Bhagawa (NRCP) is depicted in Figure 1. There are total 127 identical set of genes identified across 16 cp genomes, of which 82 genes are coding for proteins, 37 genes for tRNAs, and 8 for rRNAs, respectively. Six protein-coding genes (*rps7*, *rps12*, *rpl2*, *rpl23*, *ndhB*, and *ycf2*), seven tRNA genes (*trnM*-CAU, *trnL*-CAA, *trnN*-GUU, *trnV*-GAC, *trnE*-UUC, *trnA*-UGC, and *trnR*-ACG), and four rRNA genes (*4.5S*, *5S*, *16S*, and *23S*) are located at the IR regions (Figure 1).

**TABLE 2:** Summary of complete chloroplast genomes of 16 pomegranate genotypes.

	*Punica granatum* (*Pg*)															
	Cultivars													Wild		
Parameters	SL	SA	B	DF	RN	SB	M	W	A	R	J	G	GR	1201	1181	8718
Genome Size (bp)	158641	158641	158641	158639	158638	158641	158641	158643	158641	158633	158641	158641	158662	158593	158593	158633
Overall GC content (%)	36.9	36.9	36.9	36.9	36.9	36.9	36.9	36.9	36.9	36.9	36.9	36.9	36.9	36.9	36.9	36.9
LSC size (bp)	89025	89025	89025	89021	89020	89025	89025	89027	89025	89014	89025	89025	89044	88976	88976	89016
IR-A size (bp)	25467	25467	25467	25467	25467	25467	25467	25467	25467	25467	25467	25467	25467	25467	25467	25467
IR-B size (bp)	25467	25467	25467	25467	25467	25467	25467	25467	25467	25467	25467	25467	25467	25467	25467	25467
SSC size (bp)	18682	18682	18682	18684	18684	18682	18682	18682	18682	18685	18682	18682	18684	18683	18683	18683
Protein coding regions (bp)	77059	77083	77083	77059	77059	77181	77083	77059	77083	77083	77059	77083	77083	77059	77083	77059
rRNA size (bp)	9043	9043	9043	9043	9043	9050	9043	9043	9043	9043	9043	9043	9043	9043	9043	9043
Number of Protein coding genes	82	82	82	82	82	82	82	82	82	82	82	82	82	82	82	82
Number of tRNA	37	37	37	37	37	37	37	37	37	37	37	37	37	37	37	37
Number of rRNA	8	8	8	8	8	8	8	8	8	8	8	8	8	8	8	8
Total Number of genes	127	127	127	127	127	127	127	127	127	127	127	127	127	127	127	127

SL, Solapur Lal; SA, Solapur Anardana; B, Bhagawa; DF, double-flowering type (ornamental)*; RN, Red Nana (ornamental)*; SB, Super Bhagawa; M, Mridula; W, Wonderful; A, Arakta; R, Ruby; J, Jyoti; G, Ganesh; GR, Gul-e-Shah Red; 8718, IC-318718.

The protein-coding genes in all the 16 cp genomes constituted similar genes as shown for “Bhagawa” in Figure 1 (Supplementary Figure 1), that is, five genes for photosystem I (*psaA*, *psaB*, *psaC*, *psaI*, and *psaJ*), 14 genes for photosystem II (*psbA*, *psbB*, *psbC*, *psbD*, *psbE*, *psbF*, *psbI*, *psbJ*, *psbK*, *psbM*, *psbN*, *psbT*, *psbZ*, and *ycf3*), six genes for cytochrome b/f complex (*petA*, *petB*, *petD*, *petG*, *petL*, and *petN*), six genes for ATP synthesis (*atpA*, *atpB*, *atpE*, *atpF*, *atpH*, and *atpI*), 12 genes for NADH-dehydrogenase (*ndhA*, *ndhB*, *ndhB*, *ndhC*, *ndhD*, *ndhE*, *ndhF*, *ndhG*, *ndhH*, *ndhI*, *ndhJ*, and *ndhK*), one gene for rubisco (*rbcL*), four genes for DNA-dependent RNA polymerase (*rpoA*, *rpoB*, *rpoC1*, and *rpoC2*), 14 genes for ribosomal protein small subunit (SSU, *rps11*, *rps12*, *rps12*, *rps14*, *rps15*, *rps16*, *rps18*, *rps19*, *rps2*, *rps3*, *rps4*, *rps7*, *rps7*, and *rps8*), 10 genes for ribosomal proteins large subunit (LSU, *rpl14*, *rpl16*, *rpl2*, *rpl2*, *rpl20*, *rpl22*, *rpl23*, *rpl23*, *rpl33*, and *rpl36*), and 5 other genes for acetyl-CoA-carboxylase (*accD*), c-type cytochrome synthesis gene (*ccsA*), envelop membrane protein (*cemA*), protease (*clpP*), and maturase (*matK*). In our study, a total of 18 chloroplast genes contained introns, which are located in LSC, SSC, IRA, and IRB regions, 16 of which contained single intron, whereas two genes (*ycf3* and *clpP*) located in LSC have two introns (Table 3). The smallest intron in all the chloroplast genomes was determined to be for *ycf1* (30 bp) whereas the longest intron for *trnK*-UUU (2,489 bp).

**TABLE 3:** Summary of the intron and exon in genes of 16 chloroplast (cp) genomes.

**Gene**	**Location**	**Exon I**	**Intron I**	**Exon II**	**Intron II**	**Exon III**
*trnK*-UUU	LSC	37	2489	35		
*trnS*-CGA	LSC	32	719	60		
*atpF*	LSC	145	760	410		
*rpoC1*	LSC	432	749	1608		
*ycf3*	LSC	124	765	230	727	153
*trnL*-UAA	LSC	35	517	50		
*trnC*-ACA		38	582	56		
*clpP*	LSC	71	809	294	608	226
*petB*	LSC	6	779	642		
*ndhF*	SSC	2213	1104	100		
*ndhA*	SSC	553	1049	539		
*ycf1*	SSC	952	30	3308		
*trnA*-UGC	SSC	37	803	36		
*trnE*-UUC	SSC	32	949	40		
*ndhB*	IRA	775	683	758		
*trnE*-UUC	IRA	32	949	40		
*trnA*-UGC	IRA	37	803	36		
*ndhB*	IRB	775	683	758		

### Comparative Plastid Genome Analysis

**FIGURE 2:**
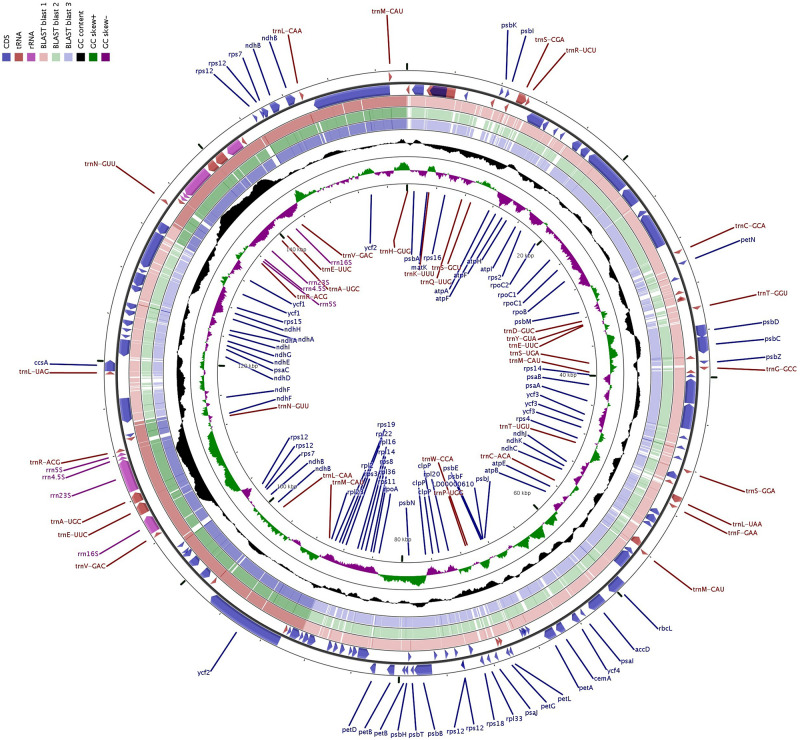
CGView: Comparative genome analyses of chloroplast (cp) genomes of related plants of the family Lythraceae.

We compared the complete cp genome of “Bhagawa” with other reported five cp genomes, i.e., “Bhagawa (R),” “Helow,” “Tunisia,” and *Lagerstroemia* (NC_034662.1 and NC_031414.1). The genome size varied from ∼152.4 kb for *Lagerstroemia* species to ∼158.6 for four *Punica* genotypes. We observed a high level of similarity for genome features between previously sequenced “Bhagawa (R)” with a newly sequenced “Bhagawa (NRCP)” cp genome. However, our assembly could reveal more information with respect to the total number of genes (127), protein coding genes 82, rRNA (8), and tRNA (37) genes compared with the previously reported “Bhagawa (R)” genome. It was also interesting to note that all *Punica* genotypes revealed a higher genome (∼158.6 kb), LSC (∼89.0 kb), and SSC (∼18.6 kb) sizes compared with ∼152. 3, 84, and 16.9 kb, respectively, in *Lagerstroemia* species. With respect to the total number of genes (112–131), protein coding genes (78–86), and genes with introns (11–18), higher variability was observed within *Punica* genomes compared with *Lagerstroemia* species (∼130 total genes, 85 protein-coding genes, and 13 genes with introns). However, GC content (37.6%) and IR size (∼25.7 kb) were slightly higher in *Lagerstroemia* species compared with GC (36.9%) and IR (∼25.5 kb) for *Punica* genomes. Chloroplast genomic structural features were also compared through a Circos graph by taking “Bhagawa” cp as a reference genome in comparison with other sequenced genomes (Figure 2). For this, BLASTn was performed for CDS to identify conserved regions between the “Bhagawa” and other genomes. The graph showed higher sequence similarity between the reference genome with the other cp genomes based on primary sequence as depicted in the Figure 2, where the first two rings (outermost, rings 1 and 2) depicted features from forward and reverse strands, respectively, from the primary sequence read file of “Bhagawa (NRCP)”; ring 3 for “Tunisia” (NC_035240.1); ring 4 for *L. intermedia* (NC_034662.1); ring 5 for *L. speciosa* (NC_031414.1), and rings 6 and 7 for GC content; and GC skew, respectively. The darker regions in the circles indicated the presence of multiple hits to the corresponding portion of the reference sequence.

### Structural Variant Analysis of Plastid Genomes

#### Inverted Repeat Expansion and Contraction

**FIGURE 3:**
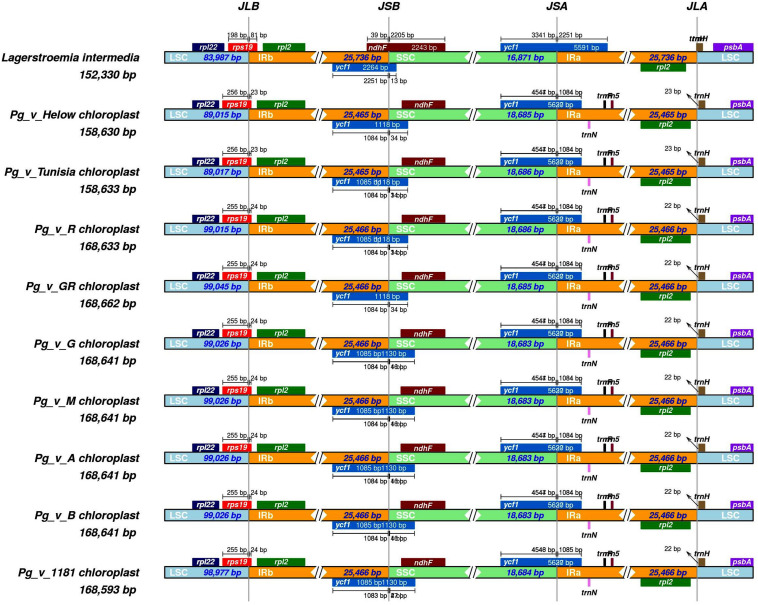
Comparison of the borders of the large single copy (LSC), small single copy (SSC), and IRs regions among representative pomegranate and *Lagerstroemia* cp genomes.

We performed a detailed comparison for borders between the IRs and single-copy regions (Table 4). The IR analysis of *L. intermedia*, “Bhagawa,” “Helow,” “Tunisia” along with six other pomegranate genotypes showed four junctions (JLA, JLB, JSA, and JSB) in between the IRs (IRa and IRb) and the two single copies (LSC and SSC) (Figure 3). The variation of up to 12 bp has been observed among *Punica* genotypes at the JSB junction. At JLB and JLA, “Tunisia” and “Helow” showed a variation of 1 bp compared with other pomegranate genotypes. However, *L. intermedia* showed considerable variation at all the four junctions, *viz.*, JLA, JLB, JSA, and JSB with respect to locations of *ycf1*, *nhdF*, *rps19*, and *trnH* genes as depicted in Figure 3.

**TABLE 4:** Comparison of the “Bhagawa (NRCP)” chloroplast genome with other genotypes.

	*Punica granatum*				Other species	
Parameters	Bhagawa (NRCP)	Bhagawa (R)	Helow	Tunisia (NC035240)	*L. intermedia* (NC034662)	*L. speciosa* (NC031414)
Genome Size (bp)	158641	158641	158630	158633	152330	152476
Overall GC content (%)	36.9	36.9	36.9	36.9	37.6	37.6
LSC size (bp)	89026	89026	89015	89,017	83987	84051
SSC size (bp)	18682	18682	18686	18687	16873	16979
IR size (bp)	25467	25467	25466	25465	25736	25723
Protein coding regions (bp)	77083	–	78159	78816	81300	81309
tRNA size (bp)	–	–	2816	2790	2810	2742
rRNA size (bp)	9043	–	9050	9050	9050	9046
Number of genes	127	112	131	129	130	129
Number of protein coding genes	82	78	86	84	85	85
Number of rRNA	8	4	8	8	8	8
Number of tRNA	37	30	37	37	37	36
Genes with introns	18	–	11	11	13	13

### Plastid Gene-Specific Indels/SNP Markers

**FIGURE 4:**
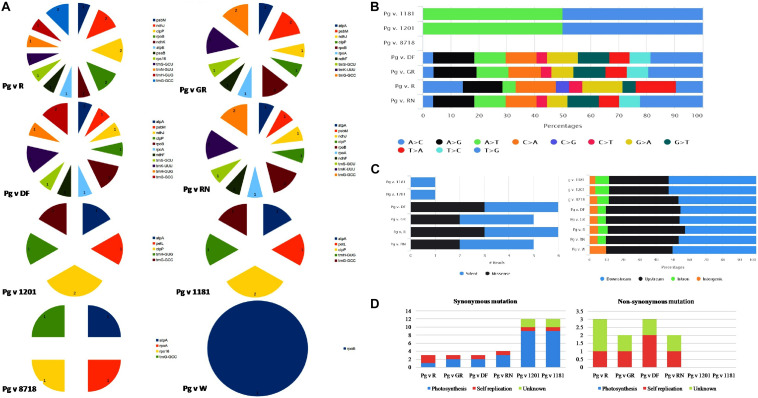
**(A)** Frequency of indels, **(B)** SNP substitutions, **(C)** SNP count by functional class, and distribution of SNPs across cp genome. **(D)** Frequency of synonymous and non-synonymous mutations.

Structural variant analysis revealed that out of 15 cp genomes that are compared with the reference “Bhagawa (NRCP)” genome, only eight genotypes had variations (Figure 4A). A maximum of 43 variants (16 indels and 27 SNPs) were identified between “Bhagawa” and Pg v. DF, followed by 42 each between “Bhagawa” and Pg v RN (15 Indels and 27 SNPs) and “Bhagawa” and Pg v GR (16 Indels and 26 SNPs), and 38 variants (21 SNPs and 17 indels) between “Bhagawa” and Pg v R. However, less than eight variants each (two SNPs and six indels) between “Bhagawa” and Pg v 1201 and “Bhagawa” and Pg v 1181 and four variants (four indels) between “Bhagawa” and Pg v 8718 were observed, but we could not detect any variations for remaining genotypes. Indels in conserved open reading frame with unknown function are not depicted in the figures (Supplementary File 2). It was also evident from Figure 4A that the maximum indels were observed for 17 key chloroplast genes across the genomes, i.e., *atpA*, *atpE*, *ndhF*, *ndhJ*, *ndhK*, *clpP*, *petL*, *psbM*, *psaB*, *rpoA*, *rpoB*, *rps16*, *tmG*-GCC, *tmH*-GUG, *tmN*-GUU, *tmS*-GCU, and *tmK*-UUU, respectively.

With respect to substitution rates for SNPs in the cp genomes, two wild genotypes (1181 and 1201) revealed 50% transition and transversion rates for A > T and T > G, respectively, whereas, for DF, GR, and RN, comparable proportionate substitution rates were observed with a higher rate of transversion for T > G, followed by A > G as depicted in Figure 4B. The genotype R (“Ruby”) has showed variable substitution rates for most of the SNPs compared with the rest of the genotypes (Figure 4B). Out of 15 cp genomes, six genotypes (1181, 1201, DF, GR, R, and RN) showed major SNP variations in comparison with “Bhagawa (NRCP).” For all genotypes, most of the SNP variations were observed in upstream and downstream regions (Figure 4B). It was also interesting to note that the wild genotypes (1181 and 1201) revealed 100% silent variations with no change in amino acids in comparison with “Bhagawa” cp genome. However, GR and RN showed maximum missense variations (50%), followed by GR and RN (40%) resulting in change in amino acids (Figure 4C). Furthermore, we also examined synonymous (S) and non-synonymous (NS) mutations (Figure 4D). We compared these mutations and their functional roles between “Bhagawa” and other genotypes (Figure 4D). The results indicated that for photosynthesis, all the SNPs tended to be S-SNPs in six pairs. However, with respect to self-replications, a higher frequency of NS-SNPs was noticed for DF, followed by GR, RN, and R. The NS mutations in known genes were related to *rps2*, *19*, and *ycf1*, *2*, whereas for unknown functional category, the frequency of NS-SNPs was higher in R, followed by GR, DF, and RN. Interestingly, the two wild genotypes (1201 and 1181) could not reveal any functional SNPs (NS).

### Repeat Sequence Analysis

**FIGURE 5:**
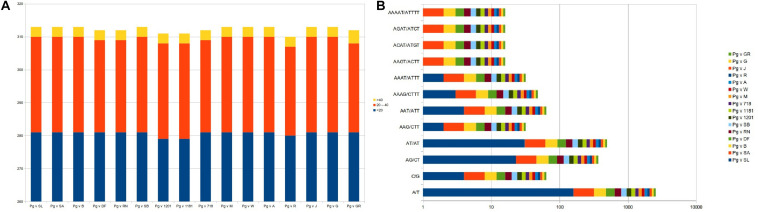
Repeat analysis and frequency distribution for **(A)** tandem repeats and **(B)** SSR motif types across the 16 plastid genomes.

Considering the role of repetitive sequences in the chloroplast genome, we performed repeat analysis. As a result, we found a total of 310–313 tandem repeats distributed across the 16 cp genomes with motif size ranging from <20 nt (∼281 numbers), 20–40 nt (∼29), and >40 nt (∼3) (Figure 5A and Supplementary Table 1).

We also surveyed for microsatellites in the 16 *Punica* cp genomes for mono- to hexanucleotide repeats (Figure 5). As a result, we identified a total of ∼233 SSRs across these genomes. The mononucleotides represented higher proportions (∼164 numbers, 70.4%) across the cp genomes, followed by di- (54, 23.18%) nucleotides, whereas, tri- (6), tetra- (4), and penta- (1) nucleotides had lesser representations in the entire genomes. With respect to frequency of SSR motif types, mononucleotides (A/T and C/G) dominated across the cp genomes, followed by di-(AT/AT and AG/CT), tri-(AAG/CTT and AAT/ATT), tetra-(AAAG/CTTT, AAAT/ATTT, AAGT/ACTT, ACAT/ATGT, and AGAT/ATCT), and pentanucleotides (AAAAT/ATTTT), respectively (Figure 5B and Supplementary Table 1).

### Phylogenetic Analysis

**FIGURE 6:**
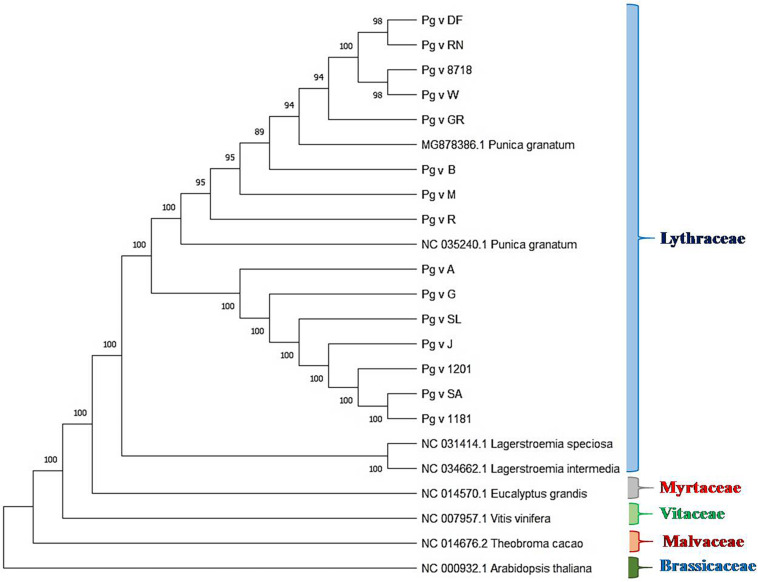
Rooted phylogenetic analysis based on cp genome sequences of different pomegranate genotypes and related plants.

We performed phylogenetic analysis to examine and determine the phylogenetic positions of sequenced pomegranate genotypes in relation to other members of the family Lythraceae and plants of other families, namely, *Arabidopsis*, cacao, grape and eucalyptus. The rooted tree clearly depicted realistic ancestry relationships among these genotypes based on the cp genome sequences as shown in Figure 6. In this tree, we observed higher bootstrap values ≥ 89 for most of the nodes. The tree clearly depicted five clades representing the families Lythraceae, Myrtaceae, Vitaceae, Malvaceae, and Brassicaceae, wherein, all the pomegranate genotypes that belonged to *Punica* and *Lagerstroemia* were grouped together as Lythraceae family with 100% bootstrap value, followed by eucalyptus (Myrtaceae), grape (Vitaceae), cacao (Malvaceae), and *Arabidopsis* (Brassicaceae). Within the Lythraceae family, all the *Punica* genotypes were clearly separated from the two *Lagerstroemia* species (NC_034662.1 and NC_031414.1) with 100% bootstrap value. Furthermore, all the *Punica* genotypes were subdivided into two subgroups, i.e., SG1 and SG2 with 100% bootstrap value. Among these, SG1 was found more diverse compared with SG2 with inclusion of unique lines such as ornamental types (double-flowered line and Nana), exotic lines (“Gul-e-Shah Red” and “Wonderful”), and previously sequenced exotic lines such as “Helow” (MG878386.1) and “Tunisia” (NC 035240_1).

## Discussion

### Comparative Plastid Genome Analysis

The comparison of 16 assembled *Punica* cp genome features revealed size variations in the plastid genomes, i.e., 158,593 bp (wild, 1201 and 1181) to 158,662 bp (cultivar, Gul-e-Shah Red), which constituted 127 genes, of which 82 coding for proteins, and 37 for tRNAs and 8 for rRNAs. These results are in best agreement with previous reports as observed in pomegranate varieties, i.e., Helow and Tunisia, with genome size (∼158.6 kb) constituting 129 to 131 genes, of which 84–86 were protein-coding genes, 37 for tRNA, and 8 for rRNA, respectively (Khan et al., 2018b; Yuan et al., 2018). The previously assembled cp genomes of other tree species also revealed nearly similar genome sizes, i.e., neem (160,737 bp, Krishnan et al., 2016), citrus (160,129 bp, Bausher et al., 2006), and heaven (Saina et al., 2018). Comparison of structural features among 16 cp genomes revealed major differences for size of LSC regions between cultivars and wild groups. The Circos graph drawn by considering “Bhagawa (NRCP)” cp as a reference genome clearly depicted all the plastid genomes having quadripartite structures, as exhibited by pomegranate (Khan et al., 2018b; Yuan et al., 2018) and other species of angiosperms, including citrus (Bausher et al., 2006), mung bean (Tangphatsornruang et al., 2009), and soybean (Asaf et al., 2017).

In the “Bhagawa (NRCP)” cp genome, we identified six protein-coding genes (*rps7*, *rps12*, *rpl2*, *rpl23*, *ndhB*, and *ycf2*), seven tRNA genes (*trnM*-CAU, *trnL*-CAA, *trnN*-GUU, *trnV*-GAC, *trnE*-UUC, *trnA*-UGC, and *trnR*-ACG), and four rRNA genes (4.5S, 5S, 16S, and 23S), which are located at the IR regions. Similarly, Yan et al. (2019b) reported on complete *de novo* assembled cp genomes of “Nana,” “Tunisia,” and “Taishanhong,” six protein-coding genes (*rps7*, *rps12*, *rpl2*, *rpl23*, *ndhB*, and *ycf2*), seven tRNA genes (*trnI-CAU*, *trnN-GUU*, *trnR-ACG*, *trnA-UGC*, *trnI-GAU*, *trnV-GAC*, and *trnL-CAA*), and four rRNA genes (4.5S, 5S, 16S, and 23S), which are located at the IR regions. The “Bhagawa (NRCP)” cp genome also revealed five genes for photosystem I, 14 genes for photosystem II, six genes for cytochrome b/f complex, six genes for ATP synthesis, 12 genes for NADH-dehydrogenase, one gene for rubisco, four genes for DNA-dependent RNA polymerase, 14 genes for ribosomal proteins SSU, 10 genes for ribosomal proteins LSU, and five other genes acetyl-CoA-carboxylase (*accD*), c-type cytochrome synthesis gene (*ccsA*), envelop membrane protein (*cemA*), protease (*clpP*), and maturase (*matK*). Similarly, Khan et al. (2018b) also observed six genes each for cytochrome b/f complex and ATP synthesis, four genes for DNA-dependent RNA polymerase, six genes for photosystem I and 15 genes for photosystem II, 11 genes for ribosomal proteins SSU, and nine genes for LSU proteins in the cp genome of Helow. Since, introns have a crucial role in the expression of specific genes when located at specific positions. Here, we report a total of 18 intron-containing genes that are located in the LSC, SSC, IRA, and IRB regions, 16 of which contained a single intron, whereas two genes (*ycf3* and *clpP*) that are located in LSC have two introns. The smallest intron in all the chloroplast genomes was determined to be for *ycf1* (30 bp) whereas the longest intron for *trnK*-UUU (2,489 bp). The results are in good agreement with Li and Zheng (2018), who observed 18 intron-containing genes in *Kadsura coccinea*; two genes, *clpP* and *ycf3*, had two introns, and *trnK*-UUU gene was found to harbor the largest intron that contained matK gene. Khan et al. (2018b) also reported 17 intron-containing genes in the pomegranate, and eight protein-coding genes had a single intron and five tRNA genes (*trnA*, *trnI*, *trnK*, *trnL*, and *trnV*), and three genes had two introns (*ycf3*, *clpP*, and *rps12*). Yan et al. (2019b) also reported 11 protein-coding genes and six tRNA genes had introns, 14 of which contain a single intron, whereas three genes, i.e., *rps12*, *ycf3*, and *clpP*, had two introns in the “Nana,” “Tunisia,” and “Taishanhong” genomes.

Furthermore, taking “Bhagawa (NRCP)” as a reference, the cp genome features were compared with other *Punica* [“Bhagawa (R),” “Helow,” and “Tunisia”] and *Lagerstroemia* (NC034662 and NC031414) genotypes. We observed 6-bp differences in the sizes of all *Punica* cp genomes in comparison with *Lagerstroemia*. In contrast to this, a higher GC content (37.6%) percentage and IR size (∼25.7 kb) were observed in Lagerstroemia compared with 36.9% GC and IR ∼25.5 kb in *Punica* genomes (Gu et al., 2016, 2017; Xu et al., 2017; Khan et al., 2018b). However, markedly higher similarities were observed within species of the same genus, i.e., *Punica* and *Lagerstroemia*. Through this study, we found that “Bhagawa (NRCP)” cp genome assembly has revealed more information with respect to the total number of genes (127), protein-coding genes 82, rRNA (8), and tRNA (37) genes compared with previously reported Indian variety “Bhagawa (R)” cp genome. These variations could be due to sequencing strategies deployed and the quality of the assembly generated after using appropriate bioinformatics tools, which would certainly cause such variations in the cp genome sizes (Cronn et al., 2008; Gallaher et al., 2018; Raime and Remm, 2018).

### Structural Variant Analysis and Cytoplasmic Markers

Structural variations in cp genomes are known to affect the genome size variations. These can be used to study the genome evolution and phylogenetic classification studies among plant lineages. Although the genome size and overall genomic structure, including gene number and gene order, are well conserved, IR expansion/contraction is very common in plant cp genomes (Kim and Lee, 2004). A structural variation of cp represents a major source of genetic diversity; therefore, here, we tried to analyze genome-wide structural variants in “Bhagawa (NRCP)” cp genome by performing pairwise comparison with the other sequenced genomes. The variations accounted due to IR expansion and contraction, indels and SNPs, and microsatellites in the cp genomes were thoroughly examined. The detailed comparison for four borders between IRs and single-copy regions revealed the major differences between species of *Lagerstroemia* and *Punica*. As suggested, although the genome size and overall genomic structures are reported to be well conserved in plants, IR expansion/contraction is common in plant cp genomes that can result in molecular diversity (Kim and Lee, 2004). In our study, all the three *Punica* genotypes, i.e., “Bhagawa (NRCP),” “Helow,” and “Tunisia” exhibited minor variations at JLB (∼1 bp), JSB (∼12 bp), and JLA (∼1 bp). Results are in agreement with those observed for Helow and Tunisia (Khan et al., 2018b). Similarly at JLB, *Lagerstroemia* species revealed (58–59 bp) a shift in *rps* 19 gene positions compared with *P. granatum* genotypes, which are well justified with the results as reported for species of *Lagerstroemia* in comparison with *Punica* (Khan et al., 2018b; Yan et al., 2019b). Overall, the striking differences were observed for sizes of LSC, SSC, Ira, and IRb regions within *Lagerstroemia* species compared with *Punica* (Khan et al., 2018b; Yan et al., 2019b). IR expansion and contraction often results in genome size variations among various plant lineages, which can be used to study the phylogenetic classification and the genome evolution among plant lineages (Yan et al., 2019b). There are three main reasons that are known to cause the diversification of the IR boundary regions: the first is intramolecular recombination, the second is the presence of multiple repeat sequences, and the third is the indels, which caused a mismatch that resulted in the upstream sequence becoming a single copy (Li and Zheng, 2018).

In this study, we have detected the maximum number of indels and SNPs between “Bhagawa (NRCP)” and Pg v DF (16 indels and 27 SNPs), followed by 42 each between “Bhagawa (NRCP)” and Pg v RN (15 indels and 27 SNPs) and “Bhagawa (NRCP)” and Pg v GR (16 indels and 26 SNPs), 38 variants (21 SNPs and 17 indels) between “Bhagawa (NRCP)” and Pg v. R, which are still less suggesting that all the 16 *Punica* cp genomes are highly conserved. However, within *Punica*, the genotypes DF, RN, GN, and GR are found most diverse in comparison with “Bhagawa,” which is also reflected from maximum indels as observed in 17 key chloroplast genes, i.e., *atpA*, *atpE*, *ndhF*, *ndhJ*, *ndhK*, *clpP*, *petL*, *psbM*, *psaB*, *rpoA*, *rpoB*, *rps16*, *tmG*-GCC, *tmH*-GUG, *tmN*-GUU, *tmS*-GCU, and *tmK*-UUU, across genotypes in comparison with Bhagawa. The “Bhagawa (NRCP)” and Pg v R showed maximum genes (12) with indels, followed by “Bhagawa (NRCP)” and Pg v DF (11 genes), “Bhagawa” and Pg v GR (10 genes), and “Bhagawa” and Pg v. RN (10 genes). Asaf et al. (2017) found that even the most conserved genome possesses some interspecific mutations, which provide an important information in analyzing the phylogenetic and genetic diversity among the species. Khan et al. (2018b) reported lesser 82 SNPs and 58 indels within *Punica* compared with *L. intermedia* (8,692 SNPs and 23,912 indels) and *Lagerstroemia floribunda* (9,104 SNPs, 24,354 indels), confirming that *P. granatum* cp genomes are highly conserved. We also examined S and NS substitutions, and found six genotypes, namely, 1181, 1201, DF, GR, R, and RN, which showed major SNP variations in comparison with Bhagawa. Furthermore, we noticed that wild genotypes (1181 and 1201) revealed 100% silent variations, and cultivars GR and RN showed maximum missense variations (50%), followed by GR and RN (40%), resulting in change in amino acids. With respect to functional role, all the SNPs tended to be S for photosynthesis in six pairs; for self-replications, higher frequency of NS-SNPs were noticed in DF, followed GR, RN, and R; for unknown functional category, frequency of NS was higher in R, followed by GR, DF, and RN. Suggested functional role of SNPs was more prominent in DF, followed by GR, RN, and R in comparison with “Bhagawa.” Yan et al. (2019b) also performed substitution analysis for 75 protein coding genes in Lythraceae family and identified that 11 genes with positively selected sites were related to genetic system or photosynthesis-related genes, suggesting that chloroplast functional genes have played vital roles during plant evolution (Dong et al., 2013). The structural variants obtained through comparative mapping of chloroplast genome sequences could lead to the development of informative cytoplasmic molecular tools for population and biogeography studies in plants (Perdereau et al., 2017).

### Repeat Analysis of Plastid Genomes

Phylogenetic studies in cp genome analysis always uses repeat sequences, since repeat sequences can be an essential source of indels and substitutions to understand the genome rearrangements (Smith, 2002; Nie et al., 2012; Yi et al., 2013; Asaf et al., 2017). The existence of repetitive sequences also suggests whether a particular region is an important hotspot for genome reconfiguration (Gao et al., 2009). Ever since, non-coding regions of cp DNA exhibit higher variation than coding regions and are an important source of variation for phylogenetic analyses of various species (Borsch and Quandt, 2009; Hajiahmadi et al., 2013). Repeat analysis in our study revealed a total of 310–313 tandem repeats distributed across *Punica* genomes with motif size range <20 nt (∼281 numbers), 20–40 nt (∼29), and >40 nt (∼3). However, Khan et al. (2018b) reported 83 repeats in the Helow genome and 85 repeats in accession #NC_035240.1, of which 31 represented tandem repeats (15–29 bp in length) in this genome. Simple sequence repeats, on the other hand, were known to have a higher mutation rate compared with other neutral DNA regions due to slipped DNA strands and have the highest diversity copy number, with known molecular markers for plant population dynamics, genetic diversity, and evolutionary studies (Rose and Falush, 1998; Huang et al., 2014; Zhao et al., 2015). In this study, a total of ∼233 SSRs were identified across cp genomes, and mononucleotides represented higher proportions (∼164 numbers, 70.4%), followed by di- (54, 23.18%) nucleotides, whereas Khan et al. (2018b) identified a total of 175 SSRs in Helow and #NC_035240.1 accessions, with trinucleotides (*n* = 62) dominating followed by dinucleotides (*n* = 54) and mononucleotides (*n* = 50). SSR diversity in the cp genome can be utilized as molecular markers for plant population dynamics, genetic diversity, and evolutionary studies (Zhao et al., 2015; Khan et al., 2018b). In recent years, many researchers used chloroplast genome for barcoding to provide more information with increased resolution at lower plant taxonomic levels. Therefore, chloroplast microsatellite loci were found highly informative to investigate the genetic diversity in Iranian pomegranate genotypes (Li et al., 2015; Coissac et al., 2016).

We also analyzed the frequency distribution for different SSR motif types across cp genomes. We found that A/T and C/G were more prominent, followed by di-(AT/AT and AG/CT) and tri-(AAG/CTT and AAT/ATT) motifs. These results are in good agreement with the previous results as observed in Helow and Accession #NC_035240.1; the percentage of “A motif” was highest (81.7%), followed by A/T and A/G, and AAG/AAT motifs in the cp genomes (Khan et al., 2018b). We also noticed that the tetra- (AAAG/CTTT, AAAT/ATTT, AAGT/ACTT, ACAT/ATGT, and AGAT/ATCT) and pentanucleotide motifs (AAAAT/ATTTT) are mainly dominated by “A” or “T” at an increased level in our study, which reflected the biased base composition with A–T richness in the cp genomes (Zhang et al., 2011; Yi et al., 2013). These results suggested that SSRs in the cp genomes are controlled by “A” or “T” mononucleotide repeats (Qian et al., 2013; Asaf et al., 2016).

### Phylogenetic Relationships

Chloroplast genome sequences have been very widely used to reconstruct plant phylogenies (Goremykin et al., 2015; Sun et al., 2016). Here, we report phylogenetic analysis based on the sequenced *Punica* cp genomes in relation to reported *Punica* (MG878386.1), *Lagerstroemia* species (NC_034662.1 and NC_031414.1), and other species such as *Arabidopsis*, cacao, grape, and eucalyptus. Rooted phylogenetic tree clearly revealed five clades representing Lythraceae, Myrtaceae, Vitaceae, Malvaceae, and Brassicaceae with high bootstrap values, where all the sequenced *Punica* genotypes were grouped along with the *Lagerstroemia* genus as a separate clade Lythraceae. Yan et al. (2019a) also reported the formation of distinct Lythraceae clades based on the phylogenetic analysis of cp genomes belonging to different families. Within Lythraceae, we observed that all the *Punica* were clearly a separation of the *Lagerstroemia* species with high bootstrap support as that of previous reports (Khan et al., 2018b, Yan et al., 2019a). Interestingly, our study also revealed that Lythraceae is taxonomically closer to the Myrtaceae family followed by Vitaceae, Malvaceae, and Brassicaceae. This has reconfirmed the earlier findings as observed through comparative chloroplast genome studies in pomegranate (Khan et al., 2018b; Yan et al., 2019a). Furthermore, Qin et al. (2017) also revealed that the pomegranate genome has much higher sequence similarity with the eucalyptus genome. In the phylogenetic tree, all the *Punica* genotypes were subdivided into two subgroups, and SG1 was found more diverse with the inclusion of ornamental types (double-flowered line and Red Nana), exotic lines (“Gul-e-Shah Red” and “Wonderful”), and previously sequenced exotic lines “Helow” and “Tunisia.” Furthermore, we also observed high nucleotide diversity at indel/SNP level in “DF,” “RN,” and “GR” in comparison with “Bhagawa (NRCP)” in this group. The diversity was very pertinent from the fact that both “DF” and “RN” are morphologically very contrasting to that of “Bhagawa,” as the DF seldom bears fruits due to petaloidy, and “RN” is dwarf in nature and bears miniature fruit with no commercial value for table purposes. The SG2 constituted mainly the cultivars “Arakta,” “Ganesh,” “Solapur Lal,” “Jyoti,” and “Solapur Anardana” and two wild types (IC-1201 and IC-1181). In this group, we found that the variety “Solapur Anardana” was distinctly closer to the wild types “IC-1201” and “IC-1181” as the fruit of “Solapur Anardana” is also highly acidic as found in case of the wild types “IC-1201” and “IC-1181.” Similarly, “Solapur Lal” was also grouped along with the wild types and has high genetic vigor as that of wild accessions. The variety “Ganesh” is one of the parents of “Solapur Lal,” which also might be the reason of their clustering in SG2. The variety “Ganesh” had a pedigree relationship with “Arakta” in this group as a female parent suggesting maternal inheritance of chloroplasts during varietal development. These findings would offer a valuable information on understanding the evolutionary history of the family Lythraceae through availability of valuable genomic resources.

## Conclusion

Through this study, we tried to report large-scale sequencing and assembling of cp genomes of cultivars, ornamental, and wild types of pomegranate. We successfully developed first-hand information on cp genomics resources through comparative cp genome analysis leading to discovery of many varietal specific cytoplasmic variations like SNP/indels and SSR markers. The research findings could leverage the future genomic-assisted breeding in pomegranate through genetic, taxonomical, and phylogenetic studies. This comprehensive study could be highly useful in understanding the phylogenetic evolutionary history of pomegranate genotypes across the related plant species.

## Data Availability Statement

The datasets presented in this study can be found in online repositories. The names of the repository/repositories and accession number(s) can be found in the article/Supplementary Material and doi: 10.5281/zenodo.4497146.

## Author Contributions

NS, PP, RP, and SP designed the research experiments and drafted and finalized the manuscript with the assistance of KB, JS, RKP, and UR. NS, PN, and PP performed the secondary analysis. All authors contributed to the article and approved the submitted version.

## Conflict of Interest

The authors declare that the research was conducted in the absence of any commercial or financial relationships that could be construed as a potential conflict of interest.

## Publisher’s Note

All claims expressed in this article are solely those of the authors and do not necessarily represent those of their affiliated organizations, or those of the publisher, the editors and the reviewers. Any product that may be evaluated in this article, or claim that may be made by its manufacturer, is not guaranteed or endorsed by the publisher.

## Footnotes

1
http://doi.org/10.5281/zenodo.4497146

## Supplementary Material

Supplementary Figure 1 Genome map of all cp genomes of pomegranate.

Supplementary Table 1 Frequency and distribution of repeats and SSR motifs in *Punica granatum* cp genomes.

Supplementary File 1 GenBank files of all pomegranate chloroplast genomes.

Supplementary File 2 Mutations in all *Punica* cp genomes.
