# Corrigendum: Chloroplast genome sequencing, comparative analysis, and discovery of unique cytoplasmic variants in pomegranate (*Punica granatum* L.)

**DOI:** 10.3389/fgene.2022.1047979

**Published:** 2022-10-17

**Authors:** Nripendra Vikram Singh, Prakash Goudappa Patil, P. Roopa Sowjanya, Shilpa Parashuram, Purushothaman Natarajan, Karuppannan Dhinesh Babu, Ram Krishna Pal, Jyotsana Sharma, Umesh K. Reddy

**Affiliations:** ^1^ ICAR-National Research Centre on Pomegranate (NRCP), Solapur, India; ^2^ Gus R. Douglass Institute and Department of Biology, West Virginia State University, West Virginia, WV, United States

**Keywords:** chloroplast, plastid genome, pomegranate, phylogenetic, sequence diversity

In the original article, there was an error in Table 2. Parameters “Gene Size(bp)” & “tRna size (bp) values” were not relevant and have been removed. The corrected table appears below.

**TABLE 2:** Summary of complete chloroplast genomes of 16 pomegranate genotypes.

Parameters	*Punica granatum* (*Pg*)															
	Cultivars													Wild		
	SL	SA	B	DF	RN	SB	M	W	A	R	J	G	GR	1201	1181	8718
Genome Size (bp)	158,641	158,641	158,641	158,639	158,638	158,641	158,641	158,643	158,641	158,633	158,641	158,641	158,662	158,593	158,593	158,633
Overall GC content (%)	36.9	36.9	36.9	36.9	36.9	36.9	36.9	36.9	36.9	36.9	36.9	36.9	36.9	36.9	36.9	36.9
LSC size (bp)	89,025	89,025	89,025	89,021	89,020	89,025	89,025	89,027	89,025	89,014	89,025	89,025	89,044	88,976	88,976	89,016
IR-A size (bp)	25,467	25,467	25,467	25,467	25,467	25,467	25,467	25,467	25,467	25,467	25,467	25,467	25,467	25,467	25,467	25,467
IR-B size (bp)	25,467	25,467	25,467	25,467	25,467	25,467	25,467	25,467	25,467	25,467	25,467	25,467	25,467	25,467	25,467	25,467
SSC size (bp)	18,682	18,682	18,682	18,684	18,684	18,682	18,682	18,682	18,682	18,685	18,682	18,682	18,684	18,683	18,683	18,683
Protein coding regions (bp)	77,059	77,083	77,083	77,059	77,059	77,181	77,083	77,059	77,083	77,083	77,059	77,083	77,083	77,059	77,083	77,059
rRNA size (bp)	9,043	9,043	9,043	9,043	9,043	9,050	9,043	9,043	9,043	9,043	9,043	9,043	9,043	9,043	9,043	9,043
Number of Protein coding genes	82	82	82	82	82	82	82	82	82	82	82	82	82	82	82	82
Number of tRNA	37	37	37	37	37	37	37	37	37	37	37	37	37	37	37	37
Number of rRNA	8	8	8	8	8	8	8	8	8	8	8	8	8	8	8	8
Total Number of genes	127	127	127	127	127	127	127	127	127	127	127	127	127	127	127	127

SL, Solapur Lal; SA, Solapur Anardana; B, Bhagawa; DF, double-flowering type (ornamental)*; RN, Red Nana (ornamental)*; SB, Super Bhagawa; M, Mridula; W, Wonderful; A, Arakta; R, Ruby; J, Jyoti; G, Ganesh; GR, Gul-e-Shah Red; 8718, IC-318718.

There was also an error in Table 4. For Parameters “tRNA size (bp)” and “rRNA size (bp)”, the value for “Bhagawa (NRCP)” was modified from “9043” and “10606” to “ ‐ ” and “9043” respectively. The corrected table appears below.

**TABLE 4:** Comparison of the “Bhagawa (NRCP)” chloroplast genome with other genotypes.

Parameters	*Punica granatum*			Other species		
	Bhagawa (NRCP)	Bhagawa (R)	Helow	Tunisia (NC035240)	*L. intermedia* (NC034662)	*L. speciosa* (NC031414)
Genome Size (bp)	158,641	158,641	158,630	158,633	152,330	152,476
Overall GC content (%)	36.9	36.9	36.9	36.9	37.6	37.6
LSC size (bp)	89,026	89,026	89,015	89,017	83,987	84,051
SSC size (bp)	18,682	18,682	18,686	18,687	16,873	16,979
IR size (bp)	25,467	25,467	25,466	25,465	25,736	25,723
Protein coding regions (bp)	77,083	-	78,159	78,816	81,300	81,309
tRNA size (bp)	-	-	2,816	2,790	2,810	2,742
rRNA size (bp)	9,043	-	9,050	9,050	9,050	9,046
Number of genes	127	112	131	129	130	129
Number of protein coding genes	82	78	86	84	85	85
Number of rRNA	8	4	8	8	8	8
Number of tRNA	37	30	37	37	37	36
Genes with introns	18	-	11	11	13	13

The authors apologize for this error and state that this does not change the scientific conclusions of the article in any way. The original article has been updated.

