# Supplementary figures and images for: Chloroplast Genome Sequencing, Comparative Analysis, and Discovery of Unique Cytoplasmic Variants in Pomegranate (Punica granatum L.)
Source: Front Genet. 2021 Jul 28;12:704075. doi: 10.3389/fgene.2021.704075 (PMC8356083; doi:10.3389/fgene.2021.704075)

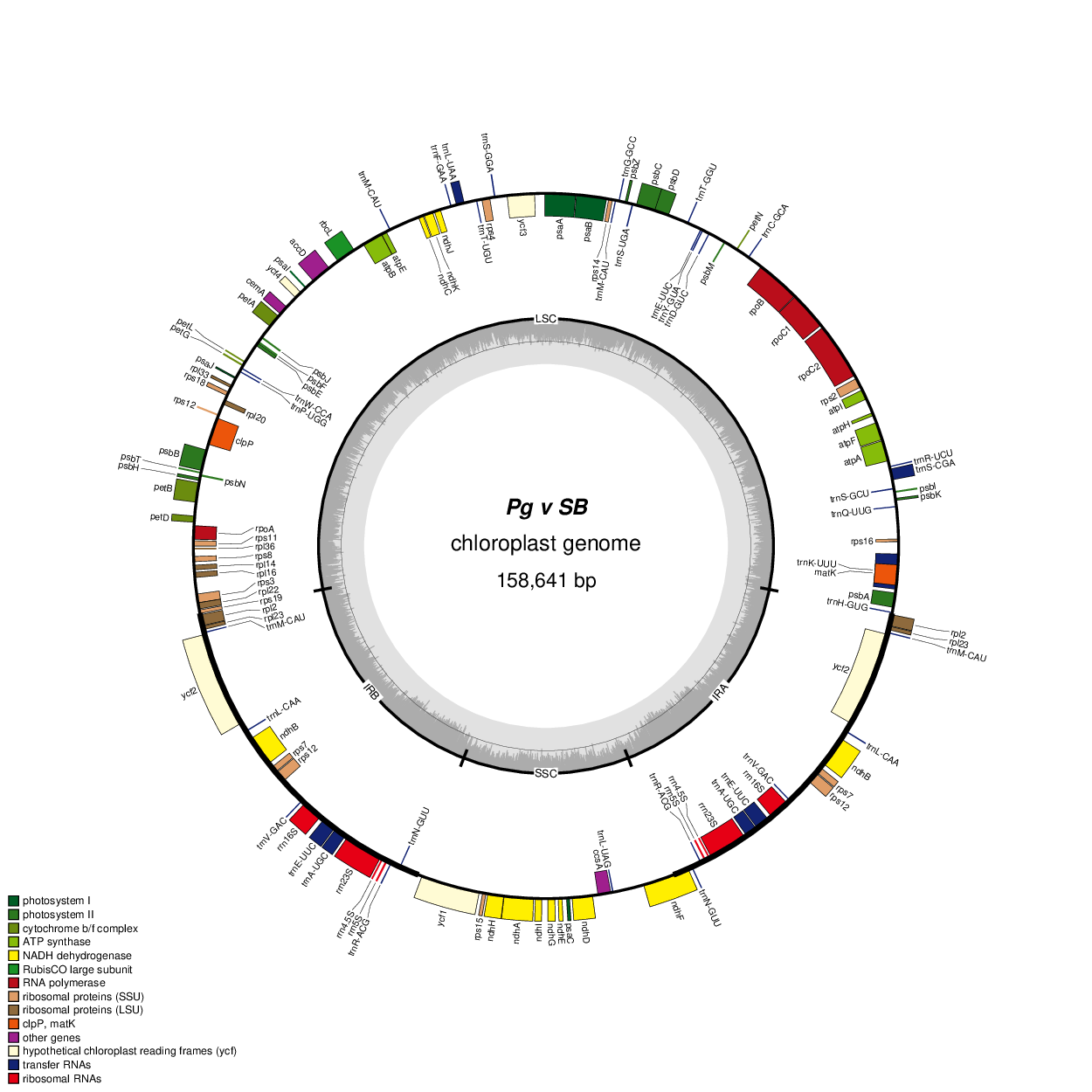


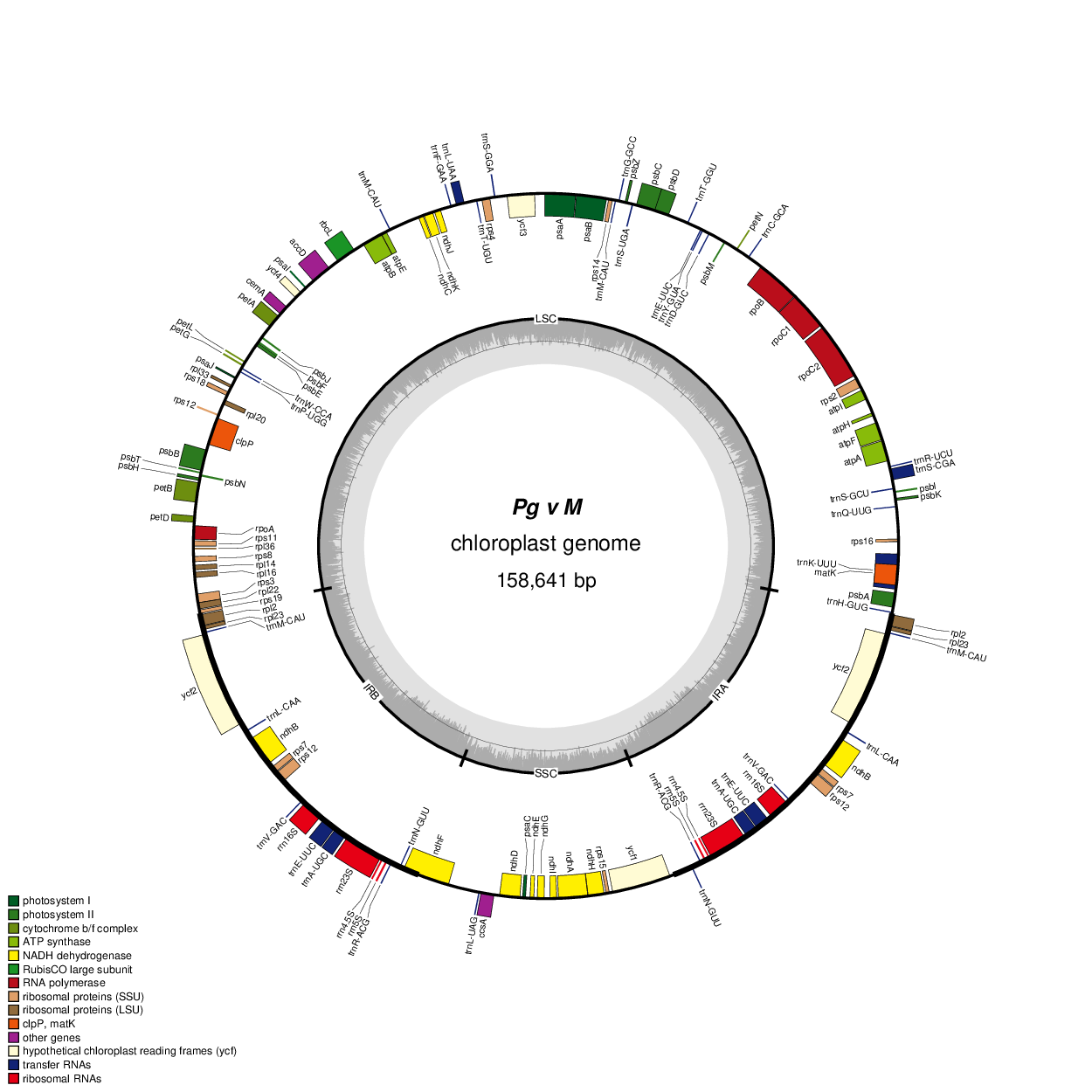


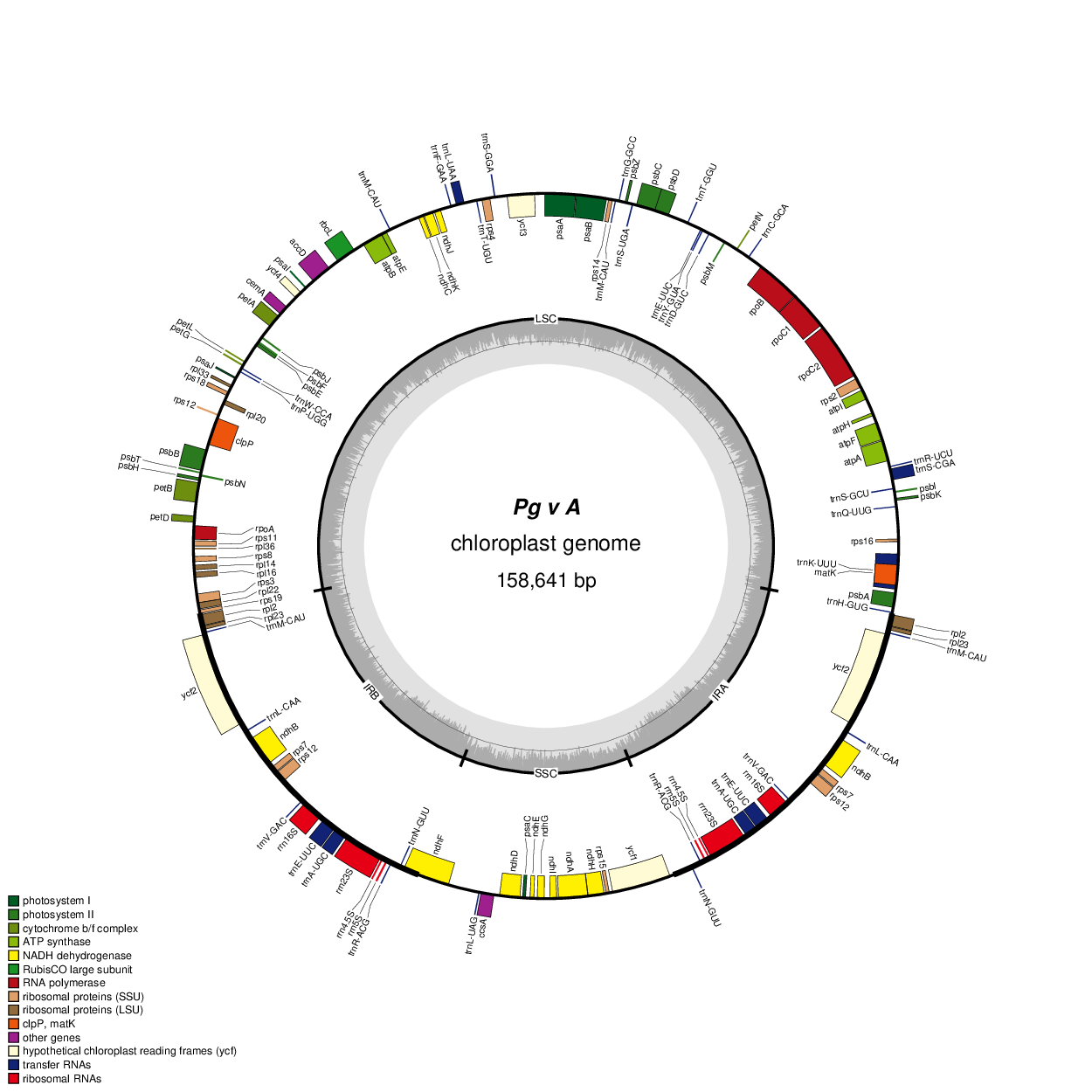


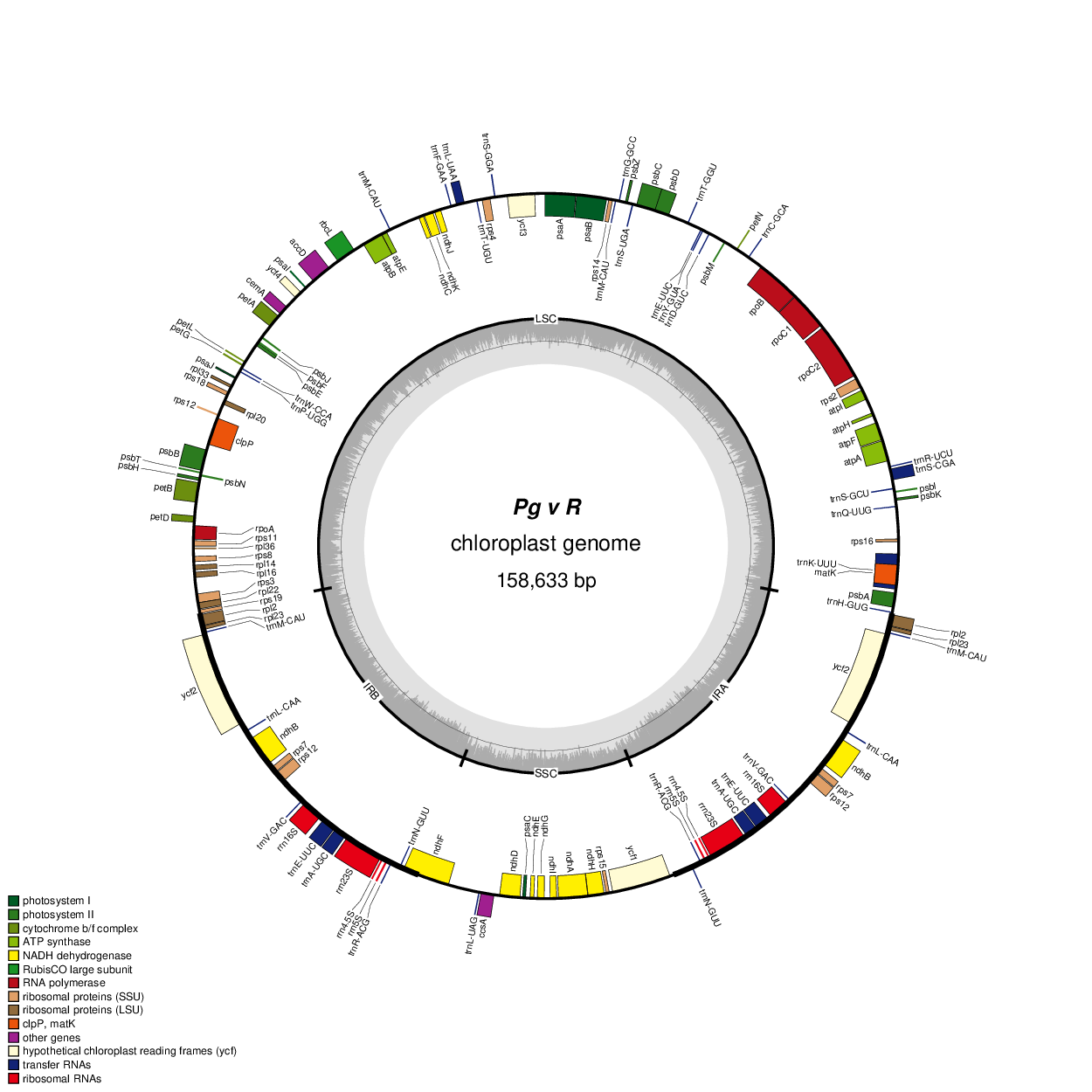


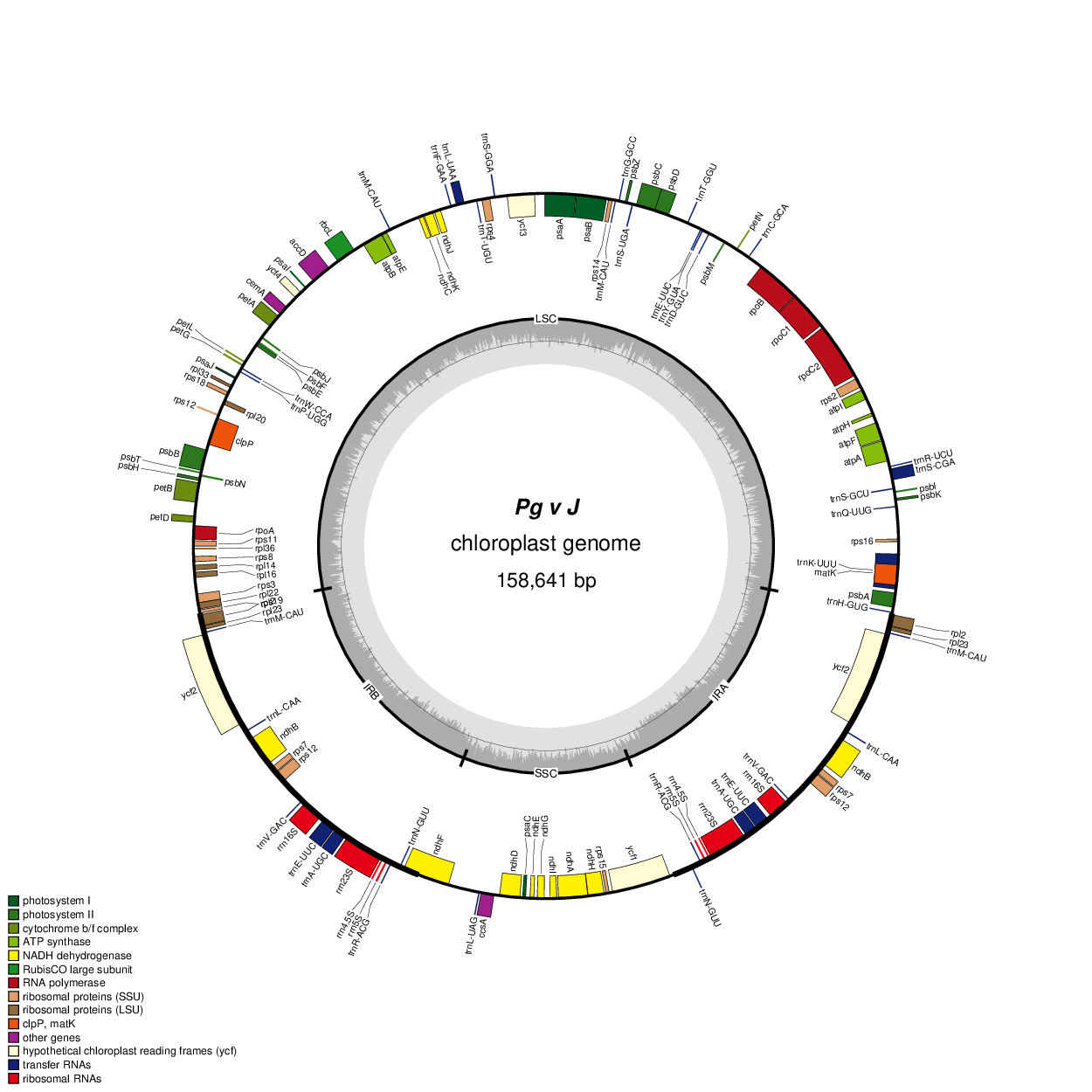


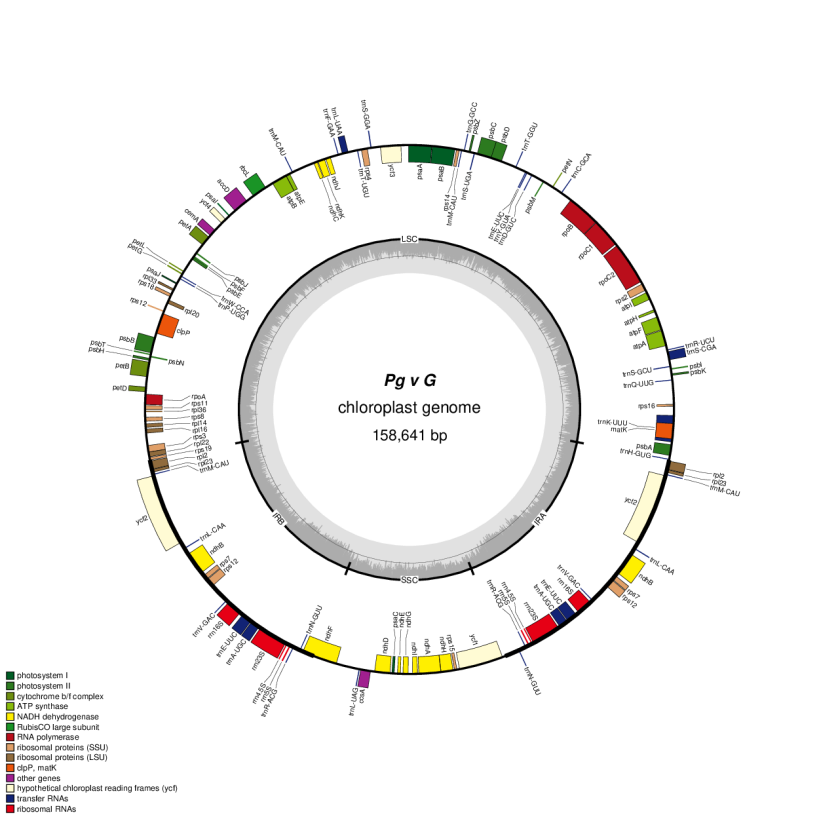


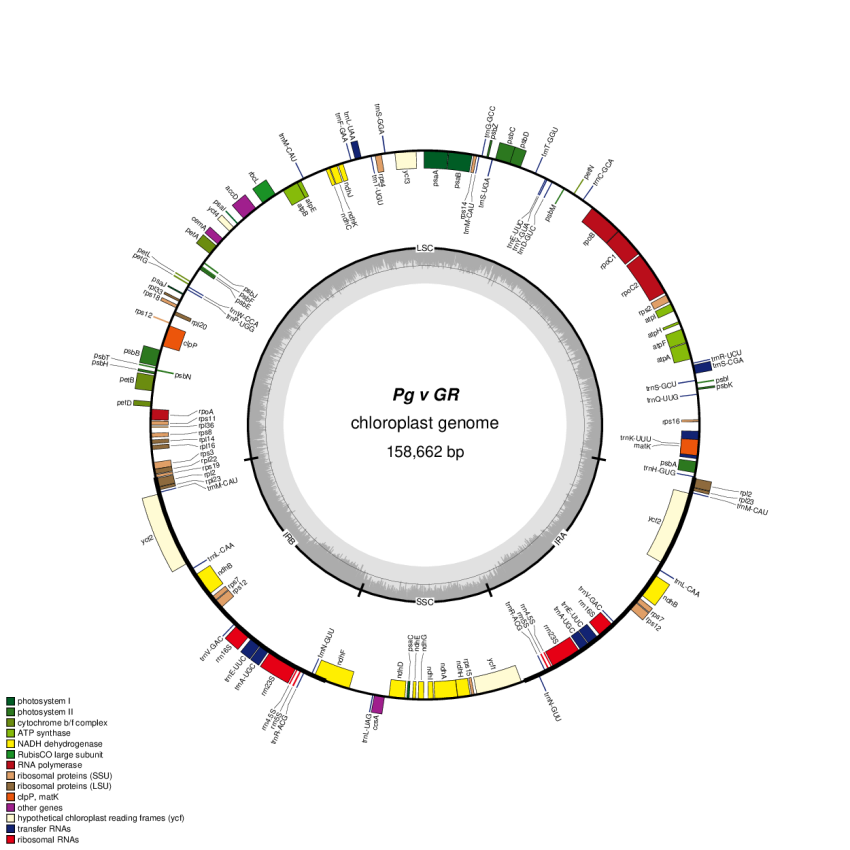


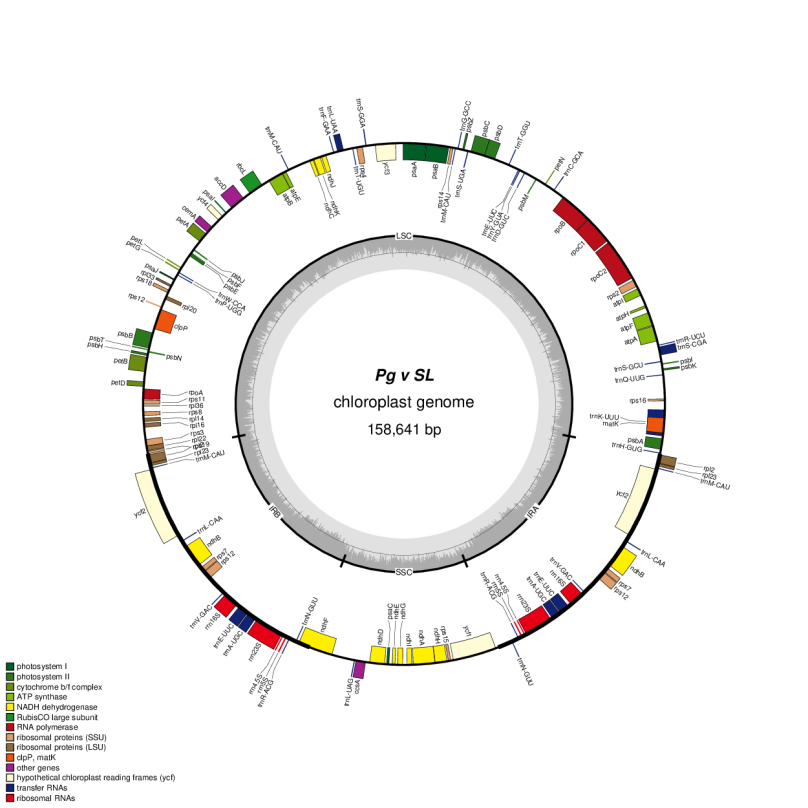


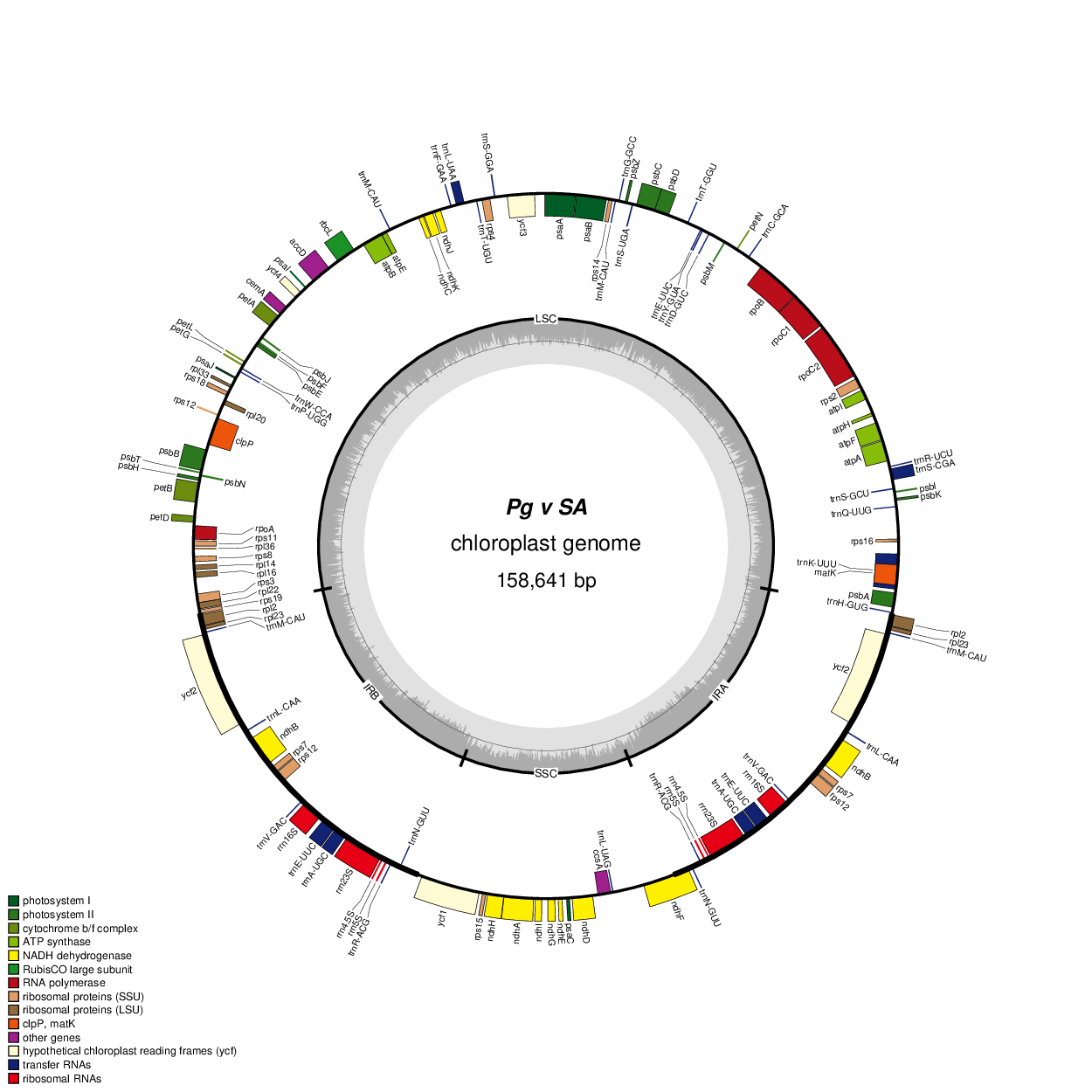


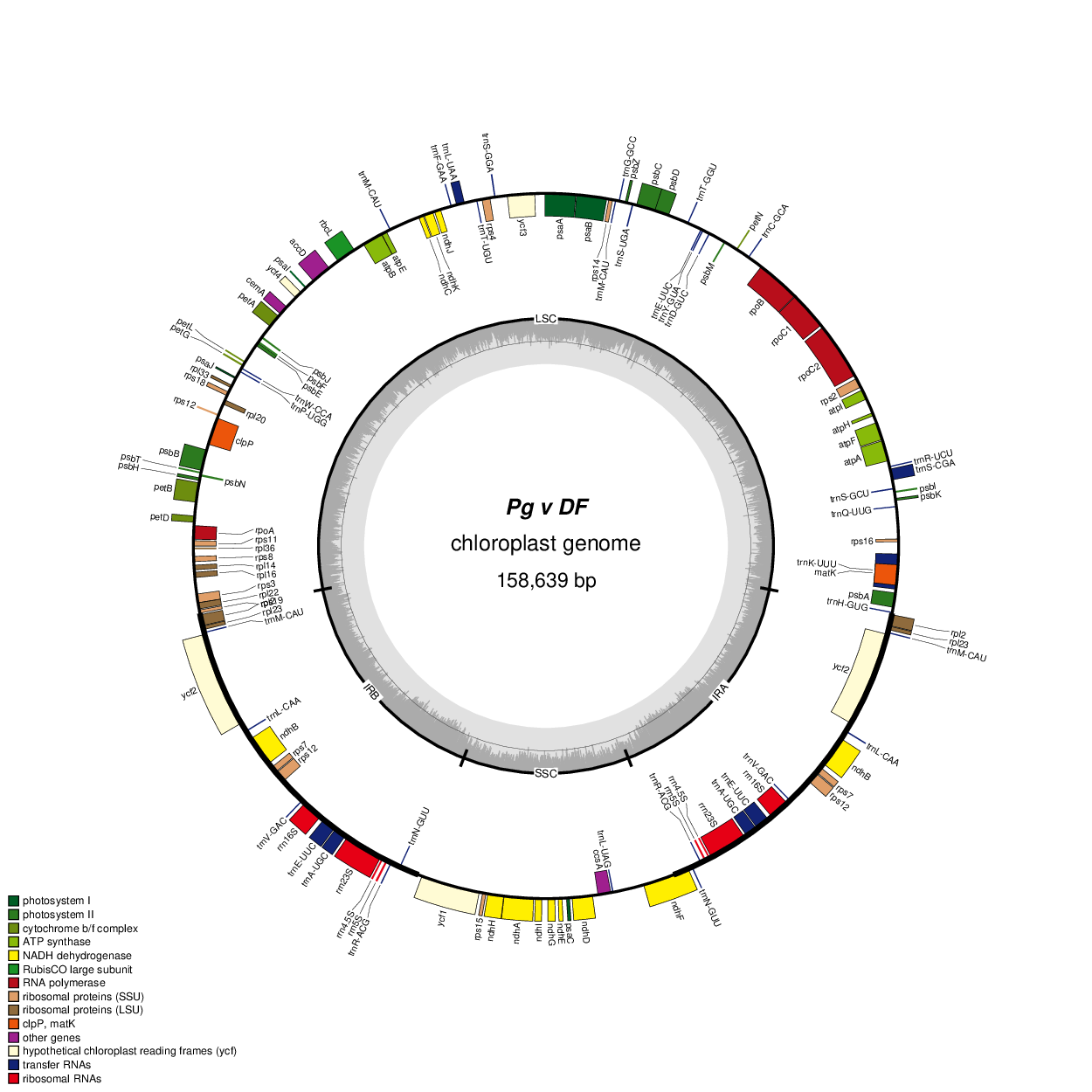


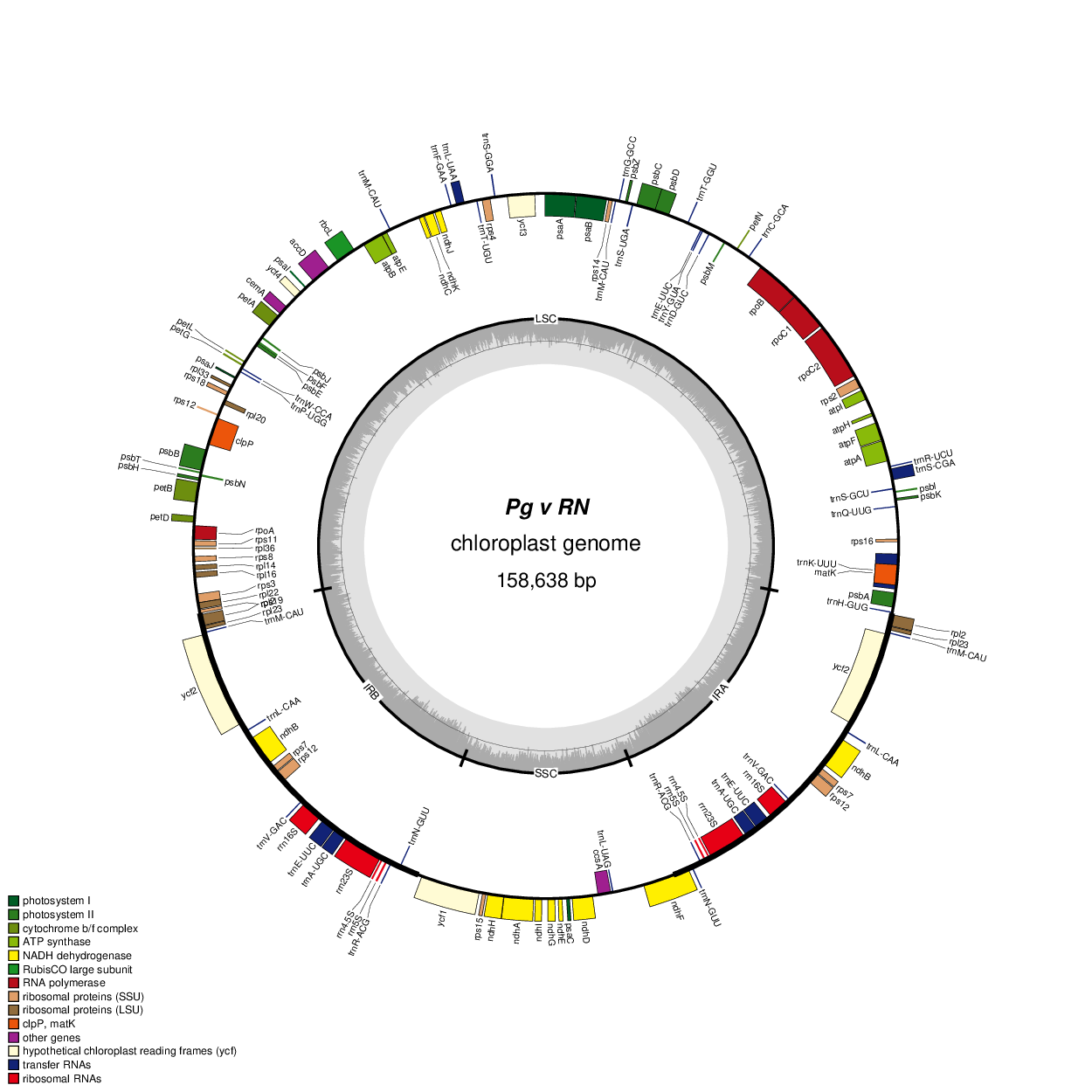


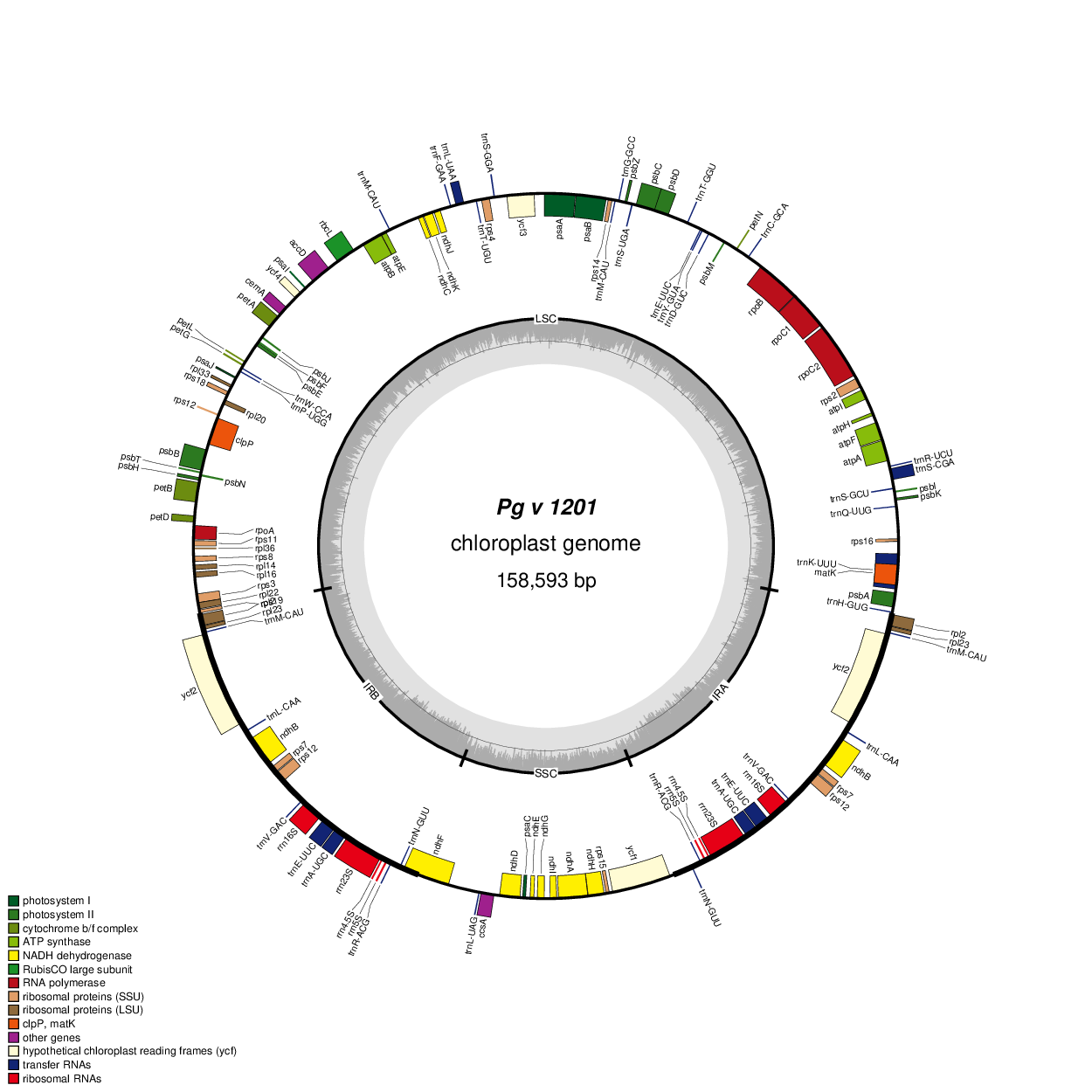


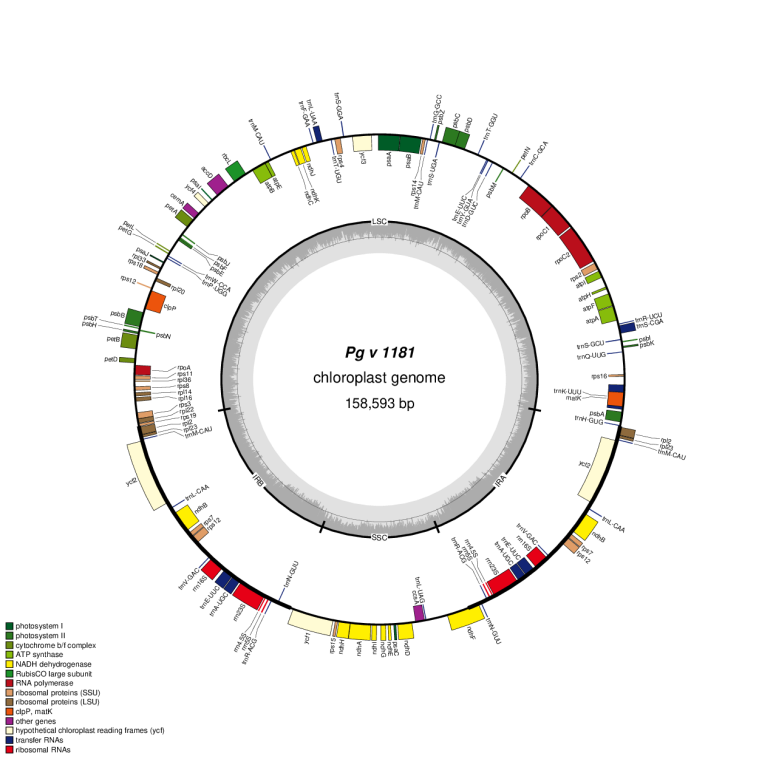


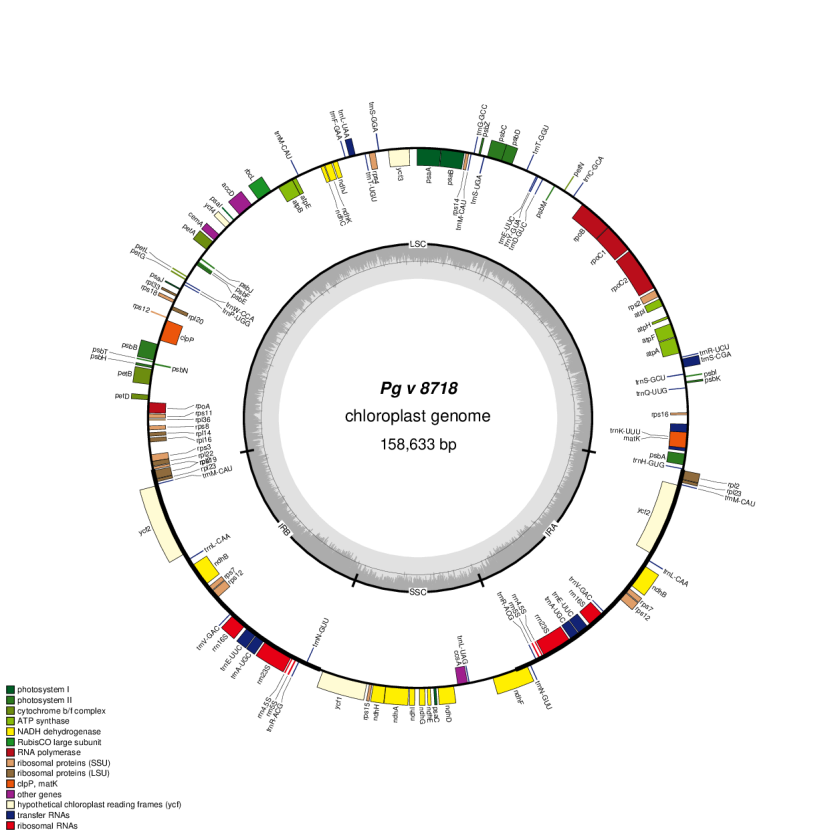


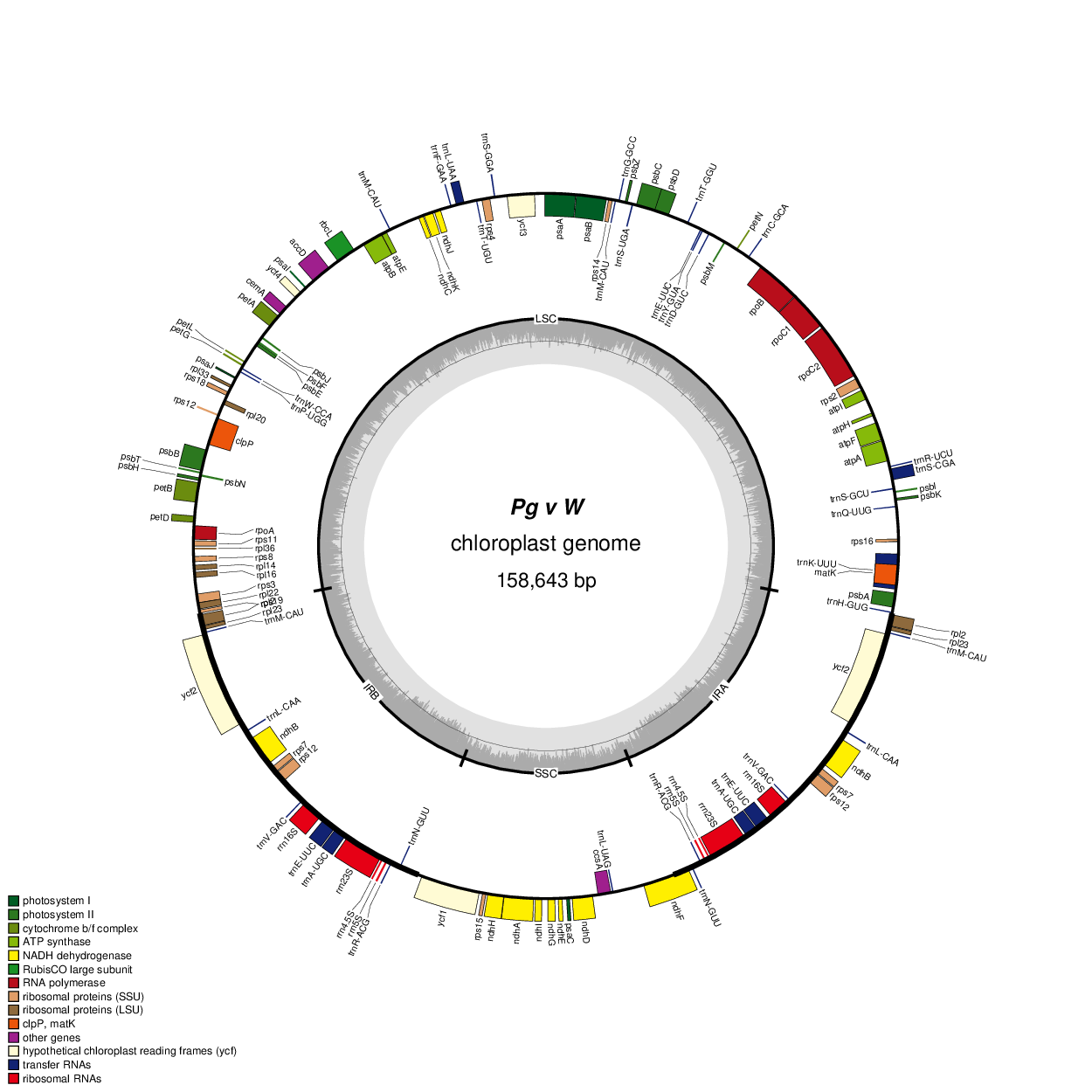

Supplement: Supplementary Figure 1 — Genome map of all cp genomes of pomegranate. [file Data_Sheet_3.docx]
