# Supplementary material for: Chloroplast Genome Sequencing, Comparative Analysis, and Discovery of Unique Cytoplasmic Variants in Pomegranate (Punica granatum L.)
Source: Front Genet. 2021 Jul 28;12:704075. doi: 10.3389/fgene.2021.704075 (PMC8356083; doi:10.3389/fgene.2021.704075)
Supplement: Supplementary Table 1 — Frequency and distribution of repeats and SSR motifs in Punica granatum cp genomes. [file Table_1.docx]

**Supplementary Table 1 Frequency and distribution of tandem repeats and SSR motifs**

(A)

| **Size Counts/GT** | **Pg v SL** | **Pg v SA** | **Pg v B** | **Pg v DF** | **Pg v RN** | **Pg v SB** | **Pg v 1201** | **Pg v 1181** | **Pg v 718** | **Pg v M** | **Pg v W** | **Pg v A** | **Pg v R** | **Pg v J** | **Pg v G** | **Pg v GR** |
| --- | --- | --- | --- | --- | --- | --- | --- | --- | --- | --- | --- | --- | --- | --- | --- | --- |
| **<20** | 281 | 281 | 281 | 281 | 281 | 281 | 279 | 279 | 281 | 281 | 281 | 281 | 280 | 281 | 281 | 281 |
| **20 – 40** | 29 | 29 | 29 | 28 | 28 | 29 | 29 | 29 | 28 | 29 | 29 | 29 | 27 | 29 | 29 | 27 |
| **>40** | 3 | 3 | 3 | 3 | 3 | 3 | 3 | 3 | 3 | 3 | 3 | 3 | 3 | 3 | 3 | 4 |
| **Total** | **313** | **313** | **313** | **312** | **312** | **313** | **311** | **311** | **312** | **313** | **313** | **313** | **310** | **313** | **313** | **312** |

(B)

(C)

| **Unit** | **Pg v SL** | **Pg v SA** | **Pg v B** | **Pg v DF** | **Pg v RN** | **Pg v SB** | **Pg v 1201** | **Pg v 1181** | **Pg v 718** | **Pg v M** | **Pg v W** | **Pg v A** | **Pg v R** | **Pg v J** | **Pg v G** | **Pg v GR** |
| --- | --- | --- | --- | --- | --- | --- | --- | --- | --- | --- | --- | --- | --- | --- | --- | --- |
| **1** | 164 | 164 | 164 | 165 | 165 | 164 | 164 | 164 | 164 | 164 | 164 | 164 | 164 | 164 | 164 | 165 |
| **2** | 54 | 54 | 54 | 54 | 54 | 54 | 54 | 54 | 54 | 54 | 54 | 54 | 54 | 54 | 54 | 54 |
| **3** | 6 | 6 | 6 | 6 | 6 | 6 | 6 | 6 | 6 | 6 | 6 | 6 | 6 | 6 | 6 | 6 |
| **4** | 8 | 8 | 8 | 8 | 8 | 8 | 8 | 8 | 8 | 8 | 8 | 8 | 8 | 8 | 8 | 8 |
| **5** | 1 | 1 | 1 | 1 | 1 | 1 | 1 | 1 | 1 | 1 | 1 | 1 | 1 | 1 | 1 | 1 |
| **Total** | **233** | **233** | **233** | **234** | **234** | **233** | **233** | **233** | **233** | **233** | **233** | **233** | **233** | **233** | **233** | **234** |

| **Repeats/ Genotypes** | **Pg v SL** | **Pg v SA** | **Pg v B** | **Pg v DF** | **Pg v RN** | **Pg v SB** | **Pg v 1201** | **Pg v 1181** | **Pg v 718** | **Pg v M** | **Pg v W** | **Pg v A** | **Pg v R** | **Pg v J** | **Pg v G** | **Pg v GR** |
| --- | --- | --- | --- | --- | --- | --- | --- | --- | --- | --- | --- | --- | --- | --- | --- | --- |
| **A/T** | 160 | 160 | 160 | 161 | 161 | 160 | 160 | 160 | 160 | 160 | 160 | 160 | 160 | 160 | 160 | 161 |
| **C/G** | 4 | 4 | 4 | 4 | 4 | 4 | 4 | 4 | 4 | 4 | 4 | 4 | 4 | 4 | 4 | 4 |
| **AG/CT** | 23 | 23 | 23 | 23 | 23 | 23 | 23 | 23 | 23 | 23 | 23 | 23 | 23 | 23 | 23 | 23 |
| **AT/AT** | 31 | 31 | 31 | 31 | 31 | 31 | 31 | 31 | 31 | 31 | 31 | 31 | 31 | 31 | 31 | 31 |
| **AAG/CTT** | 2 | 2 | 2 | 2 | 2 | 2 | 2 | 2 | 2 | 2 | 2 | 2 | 2 | 2 | 2 | 2 |
| **AAT/ATT** | 4 | 4 | 4 | 4 | 4 | 4 | 4 | 4 | 4 | 4 | 4 | 4 | 4 | 4 | 4 | 4 |
| **AAAG/CTTT** | 3 | 3 | 3 | 3 | 3 | 3 | 3 | 3 | 3 | 3 | 3 | 3 | 3 | 3 | 3 | 3 |
| **AAAT/ATTT** | 2 | 2 | 2 | 2 | 2 | 2 | 2 | 2 | 2 | 2 | 2 | 2 | 2 | 2 | 2 | 2 |
| **AAGT/ACTT** | 1 | 1 | 1 | 1 | 1 | 1 | 1 | 1 | 1 | 1 | 1 | 1 | 1 | 1 | 1 | 1 |
| **ACAT/ATGT** | 1 | 1 | 1 | 1 | 1 | 1 | 1 | 1 | 1 | 1 | 1 | 1 | 1 | 1 | 1 | 1 |
| **AGAT/ATCT** | 1 | 1 | 1 | 1 | 1 | 1 | 1 | 1 | 1 | 1 | 1 | 1 | 1 | 1 | 1 | 1 |
| **AAAAT/ATTTT** | 1 | 1 | 1 | 1 | 1 | 1 | 1 | 1 | 1 | 1 | 1 | 1 | 1 | 1 | 1 | 1 |
